# Evaluation of the impact of dental prophylaxis on the oral microbiota of dogs

**DOI:** 10.1371/journal.pone.0199676

**Published:** 2018-06-25

**Authors:** Rebecca Flancman, Ameet Singh, J. Scott Weese

**Affiliations:** 1 Department of Pathobiology, Ontario Veterinary College, University of Guelph, Guelph, Ontario, Canada; 2 Department of Clinical Studies, Ontario Veterinary College, University of Guelph, Guelph, Ontario, Canada; Boston University Henry M Goldman School of Dental Medicine, UNITED STATES

## Abstract

Periodontal disease is one of the most commonly diagnosed oral diseases in dogs and can result from undisturbed dental plaque. Dental prophylaxis is a routinely practiced veterinary procedure, but its effects on both the plaque and oral microbiota is not fully understood. The objectives of this study were to evaluate the impact of dental prophylaxis on the composition of the supragingival plaque and composite oral microbiota in clinically healthy dogs and to determine if composite sampling could be used in lieu of sampling the plaque microbiota directly. Thirty dogs received a dental prophylaxis. Supragingival plaque and composite oral samples were collected just prior to, and one week after dental prophylaxis. A subsample of 10 dogs was followed, and additional samples were collected two and five weeks post-prophylaxis. The V4 region of the 16S rRNA gene was used for Illumina MiSeq next-generation sequencing. Results demonstrate that decreases in *Treponema* as well as increases in *Moraxella* and *Neisseria* distinguished the plaque pre- and one week post-prophylaxis timepoints (all P<0.05). Within the oral microbiota, the initially dominant *Psychrobacter* (20% relative abundance) disappeared one week later (P<0.0001), and *Pseudomonas* became the dominant taxon one week after treatment (80% relative abundance, P<0.0001). A rapid transition back towards the pre-dental prophylaxis microbiota by five weeks post-treatment was seen for both niches, suggesting the canine oral microbiota is resilient. Direct comparison of the two environments yielded striking differences, with complete separation of groups. Firmicutes (40%) and Spirochaetes (22%) predominated in the plaque while Proteobacteria (58%) was predominant in the oral microbiota. Greater richness was also seen in the plaque microbiota. This study reveals that prophylaxis had a profound impact on both the plaque and oral microbiota, and the longitudinal results help elucidate the pathophysiology of periodontal disease. The results suggest that oral swabs are a poor proxy for plaque samples and highlight the need to study specific oral niches.

## Introduction

Periodontal disease is one of the most commonly diagnosed oral diseases, ranks amongst the top medical diagnoses in dogs, and can develop as a result of plaque (a bacterial biofilm) on the teeth [1–6]. Previous research an *in vitro* model for initial canine enamel colonization [5] and has examined the longitudinal changes in plaque during periodontal disease progression [4]. However, while dental prophylaxis is a routinely practiced veterinary procedure, its effects on plaque and oral microbiotas are not fully understood. It is crucial to understand how the plaque and oral ecological niches within the canine oral cavity change over time as a result of a dental prophylaxis intervention.

Additionally, plaque is not the sole ecological community within the oral cavity. In a study of humans, three distinct bacterial communities were identified in seven different oral cavity sites (buccal mucosa, keratinized gingiva, hard palate; and saliva, tongue, tonsils, throat; and sub- and supra-gingival plaques) [7]. Differences in a variety of local factors (e.g. oxygen tension, pH, mucosal surfaces) likely impact the local microbiota. The collection of an oral swab from a dogs’ mouth is a comparatively simple exercise versus the collection of canine dental plaque. It is essential to determine how the oral microbiota relates to that of plaque, as similarities and/or differences between the populations will help determine whether oral sampling can be used as a proxy for the characterization of plaque. If this were the case, sampling in future studies could be greatly facilitated. The objectives of this study were to describe the effects of dental prophylaxis on both the plaque and composite oral microbiotas of clinically healthy dogs as well as to compare the plaque and oral microbiotas to determine if composite sampling could be used in lieu of sampling the plaque microbiota directly.

## Methods

### Ethics statement

The protocol was reviewed and approved by the Sinclair Research Center Institutional Animal Care and Use Committee. Housing and husbandry of the animals was in accordance with the Animals Welfare Act and with the “Guide for the Care and Use of Laboratory Animals” (National Research Council).

### Sample collection

#### Study subjects

Thirty healthy, adult beagle dogs (5 neutered males and 25 spayed females) from a research colony were enrolled. The dogs were between 1 and 4 years of age (mean age = 2.13 y), with a mean weight of 8.87 kg (range 7.00–13.14 kg). All dogs were clinically normal, had no history of periodontal or other oral disease, and had no history of antimicrobial exposure in the preceding three months. Dogs were fed a commercial dry diet (Purina Dog Chow—Nestlé Purina PetCare Company, St. Louis, MO 63164 USA), to which they had been acclimated.

#### Sample collection and dental prophylaxis

All dogs received a manually conducted dental prophylaxis under general anesthesia on the first day of the study (Pre). Antimicrobials were not administered as part of the procedure. Prior to the procedure, supragingival plaque was collected from the lingual and buccal surfaces of the dental arcades and composite oral swabs were collected by swabbing the gums, tongue, and cheeks for 10–15 seconds. One week after the dental prophylaxis, plaque and composite oral swabs were collected in the same manner from all 30 dogs. A random subsample of 10 of the 30 dogs was also sampled in the same manner two and five weeks after dental prophylaxis. All oral samples were collected onto sterile flocked nylon-tipped BD Liquid Amies Elution swabs (Becton, Dickinson and Company, USA) and kept at 4°C. Plaque samples were initially collected using curettes, and then transferred to the swabs for transport. Samples were shipped within 48 hours of collection, by courier, to the laboratory on dry ice.

### DNA extraction, 16S rRNA gene PCR amplification, and purification

DNA extraction was performed using a commercial kit (E.Z.N.A. Forensic DNA Kit, Buccal Swabs Protocol, Omega Bio-Tek Inc., USA). DNA concentrations were measured by spectrophotometry (NanoDrop, Thermo Scientific, USA).

PCR amplification of the V4 region of the 16S rRNA gene was performed [8]. The previously designed forward primer S-D-Bact-0564-a-S-15 (5’-TCGTCGGCAGCGTCAGATGTGTATAAGAGACAGAYTGGGYDTAAAGNG-3’) and reverse primer S-D-Bact-0785-b-A-18 (5’-GTCTCGTGGGCTCGGAGATGTGTATAAGAGACAGTACNVGGGTATCTAATCC-3’) were used for the amplification [8]. These primers contained overhanging adaptors to anneal with the Illumina universal index sequencing adaptors that were added in a later PCR. The PCR protocol used was previously described [9]. After amplification, samples were refrigerated at 4°C until further processing. The PCR products were evaluated by electrophoresis in 1.5% agarose gel and visualised under UV light using the GeneGenius bioimaging system (Syngene, USA). The amplicon library was purified using Agencourt AMPure XP beads (Beckman Coulter Inc., Canada), again using the protocol previously described [9], with the exception of re-suspension in 52.5 μL of 10mM Tris buffer, pH 8.5, instead of 50 μL. Approximately 5% of the samples did not amplify and were reprocessed, after purfying the initially extracted DNA using Agencourt AMPure XP beads.

### Indexing, purification, and DNA sequencing

A second PCR was performed to attach the Illumina sequencing adaptors and dual-indexing barcodes to the amplicons. For a final volume of 40 μL, 4 μL of each purified PCR product was added to a solution containing 20 μL KAPA 2G Fast HotStart ReadyMix (KAPA Biosystems, Wilmington, Massachusetts), 9.6 μL of HyClone molecular biology-grade water (GE Healthcare Lifesciences, Hyclone Laboratories, Logan, Utah), and 3.2 μL of both Illumina forward index primer S5XX (2.5 pM/ μL) and Illumina reverse index primer N7XX (2.5 pM/ μL). A short amplification cycle was performed to anneal the index primers to the amplicons using conditions previously described [9].

A final purification procedure was performed using a previously described procedure [9], with the exception of elution into 32 μL of 10 mM Tris buffer, pH 8.5, instead of 30 μL. Then, 30 μL from each sample was transferred to a 96-well plate. 5 μL of the amplicon library was used for evaluation by electrophoresis in 1.5% agarose gel and visualised under UV light. The remaining 25 μL of each sample was sent to the University of Guelph’s Advanced Analysis Centre, for DNA sequencing on the Illumina MiSeq platform (San Diego, USA) using 2x250 chemistry.

### Sequence analysis and statistical methods

The DNA sequences were analysed using the open-source bioinformatics software package, mothur (v1.34) [10]. The mothur standard operating procedure was used [11]. Paired end reads were merged into fully overlapping reads and the sequences were aligned using the SILVA 16S rRNA gene reference database [12]. Those that were misaligned or those with ambiguous base calls, inappropriate lengths (>242 or <239 bp), long runs of homopolymers (>8 bp), and sequences corresponding to chloroplasts, mitochondria, Archaea, and Eukaryotes were removed. Chimeras were detected using uchime [13] and removed. The remaining sequences from plaque samples were binned into operational taxonomic units (OTU) at a 3% (0.03) dissimilarity level while remaining sequences from oral microbiota sequences were binned into OTUs using a closed OTU picking approach after taxonomic assignment using the Ribosomal Database Project database [14].

For both the plaque and oral microbiota sequences, the following analyses were performed. Random subsampling was performed (based on 1000 random iterations) to normalize the sequence numbers. Alpha diversity was calculated from within mothur, using the Good’s coverage, richness (Chao1), evenness (Shannon’s evenness) and diversity (inverse Simpson’s) indices. These indices were compared using the Wilcoxon test (Pre and 1Week time points) or Steel-Dwass multiple comparisons test (all study time points), with a *p-*value of ≤ 0.05 considered significant (JMP, SAS Institute Inc).

Relative abundances were calculated and 100% stacked column graphs comparing the median relative abundances of the main phyla (abundance ≥ 0.5%) were generated; 54 genera were selected *a priori* on the basis of biological significance and high relative abundance. Wilcoxon or Steel-Dwass tests were performed with Benjamini-Hochberg adjustment for False Discovery Rate. False Discovery Rate *p-*values (*P*_FDR_) of ≤ 0.05 were considered significant.

Beta diversity was measured using the Jaccard and the Yue and Clayton indices, which measure community membership (number of shared genera) and community structure (number of shared genera and their relative abundances), respectively. These were visualized by generating dendrograms (FigTree) of the relationships between the samples. Unweighted UniFrac [15] and analysis of molecular variance (AMOVA) tests were also conducted in mothur to evaluate the impact of group (day of sampling) on the microbial community membership and structure. Principal Coordinate Analysis (PCoA) was performed on both the Jaccard and the Yue and Clayton indices with results visualized using JMP.

Linear discriminant analysis effect size (LEfSe) [16] was used to evaluate the genetic sequences and to identify genera that were enriched at the various time points.

To compare the plaque and oral microbiotas directly, a combined analysis within mothur was performed, with analysis as for the individual sample types that is described above.

## Results

### Short-term effects of dental prophylaxis on the plaque and oral microbiota

#### Sequence quality

Within the plaque microbiota, sequencing generated a total of 2,981,735 reads that passed all screening tests. Sequence numbers ranged from 31,278 to 70,814 per plaque sample (mean = 49,696, median = 48,714). A total of 846 OTUs were identified, with the top five OTUs covering over 50% of total sequences. A random subsample of 31,278 sequences per sample was used to normalize the sequence numbers for the pre-dental and one week post-dental prophylaxis plaque samples, with a median Good’s coverage of 96.8%.

Similarly, within the oral microbiota, sequencing generated a total of 5,229,454 reads that passed all screening tests. Sequence numbers ranged from 33,457 to 130,865 per oral swab sample (mean = 87,158, median = 86,559). A total of 588 OTUs were identified, with the top five OTUs composing nearly 60% of total sequences. A random subsample of 33,457 sequences per sample was used to normalize the sequence numbers for the pre-dental and one week post-dental prophylaxis oral microbiota samples, with a median Good’s coverage of 99.9%.

#### Relative abundance

**Fig 1:**
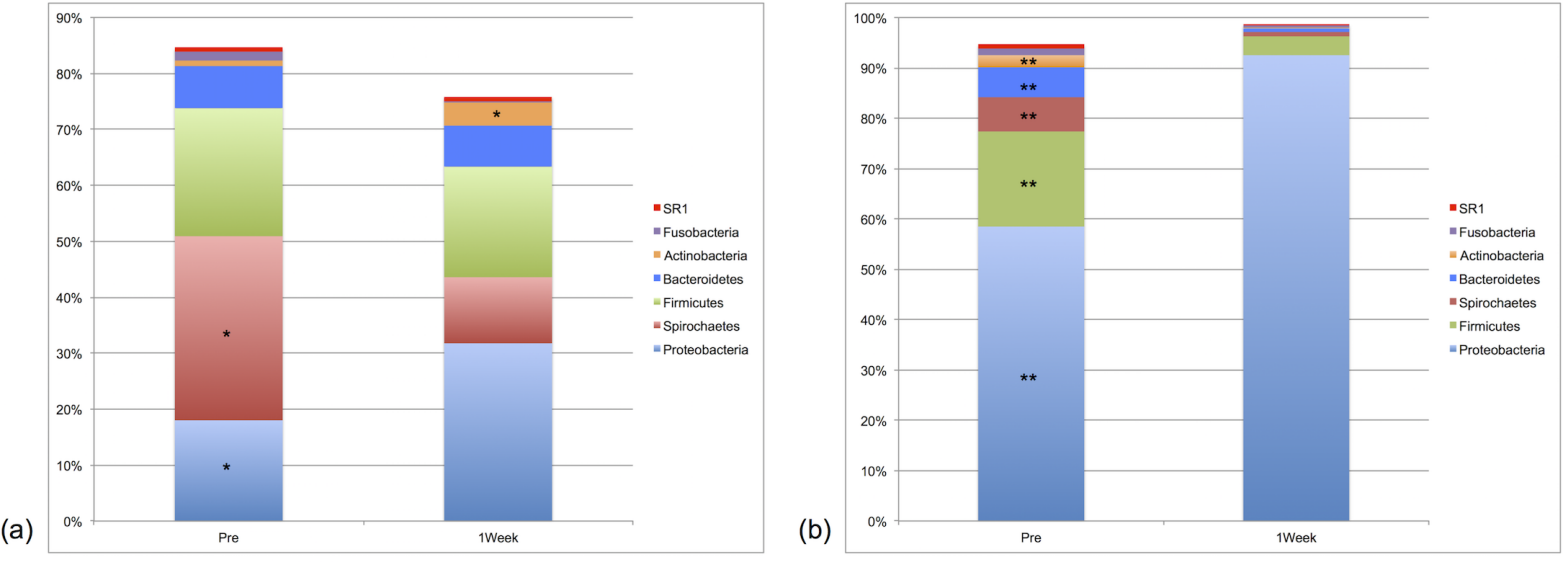
Median percent relative abundances of bacterial phyla for (a) plaque and (b) oral microbiota samples (n = 30). Pre and 1Week time points comparisons. Median relative abundance ≥ 0.5%. (a) Actinobacteria, Proteobacteria, and Spirochaetes were statistically significant between the two timepoints (*P*_FDR_ ≤ 0.003); (b) Acintobacteria, Bacteroidetes, Firmicutes, Proteobacteria, and Spirochaetes were statistically significant (*P*_FDR_ < 0.0001).

Plaque sequences were assigned to 26 different bacteria phyla, with 25 phyla identified from oral samples. While the relative abundances of these phyla differed between the niches, only five phyla (Actinobacteria, Bacteroidetes, Firmicutes, Proteobacteria, and Spirochaetes) accounted for ≥ 1% of total sequences amongst both environments (Fig 1). Within the plaque, differences in Actinobacteria, Proteobacteria, and Spirochaetes were statistically significant between the two timepoints (*P*_FDR_ ≤ 0.003). Within the oral microbiota, differences in Acintobacteria, Bacteroidetes, Firmicutes, Proteobacteria, and Spirochaetes were statistically significant (*P*_FDR_ < 0.0001).

The relative abundances, *p-*values, and false discovery rate *p-*values of 54 selected genera from the pre-dental and one week post-prophylaxis plaque and oral microbiota samples are presented in S1 and S2 Tables. For the plaque, *Treponema*, an unclassified Clostridiales, and an unclassified Peptostreptococcaceae had the highest relative abundances both prior to dental prophylaxis and one week afterwards.

Early canine oral biofilm colonizing genera *Moraxella*, and *Neisseria* (Holcombe et al. 2014) had higher relative abundances in the plaque microbiota one week after dental prophylaxis compared to baseline (*P*_FDR_ = 0.006; *P*_FDR_ = 0.03, respectively). *Actinomyces*, *Corynebacterium*, *Porphyromonas*, *Tannerella*, and *Treponema* genera that have been implicated as canine periodontal pathogens, were identified in all plaque samples. *Actinomyces*, *Corynebacterium*, and *Tannerella* all had significantly higher relative abundances one week post-dental prophylaxis than before treatment (*P*_FDR_ < 0.01), while *Treponema* had a higher relative abundance before prophylaxis (*P*_FDR_ = 0.0035). There was no statistical significant difference in the relative abundance of *Porphyromonas* between the timepoints. Two genera with zoonotic potential, *Capnocytophaga* and *Pasteurella*, were also identified in all samples and had higher relative abundances one week after dental prophylaxis (*P*_FDR_ < 0.001; *P*_FDR_ = 0.003, respectively). *Streptobacillus*, a third genus with zoonotic potential had a higher relative abundance prior to treatment (*P*_FDR_ = 0.03).

With the oral microbiota, *Psychrobacter*, an unclassified Clostridiales, *Treponema*, and an unclassified Pasteurellaceae had the highest relative abundances prior to dental prophylaxis (median relative abundance ≥ 5%) while *Pseudomonas* dominated after dental prophylaxis, being the only genus with a relative abundance of 5% or greater after prophylaxis and having a median value of 79.7% (range .47.4%–92.6%).

In addition to the *Pseudomonas*, other water-dwelling genera *Fusibacter* and *Psychrobacter* had statistically significant differences in abundance between time points (*P*_FDR_ < 0.0001), with *Fusibacter* having a higher relative abundance prior to prophylaxis and *Psychrobacter* having a higher relative abundance one week after prophylaxis. Putative canine periodontal pathogens *Actinomyces*, *Fusobacterium*, *Porphyromonas*, *Tannerella*, and *Treponema* all had significant decreases in relative abundances after dental prophylaxis (*P*_FDR_ < 0.0001), as did potential zoonotic genera *Pasteurella*, and *Streptobacillus* (*P*_FDR_ < 0.0001).

#### Alpha and beta diversity analyses

**Fig 2:**
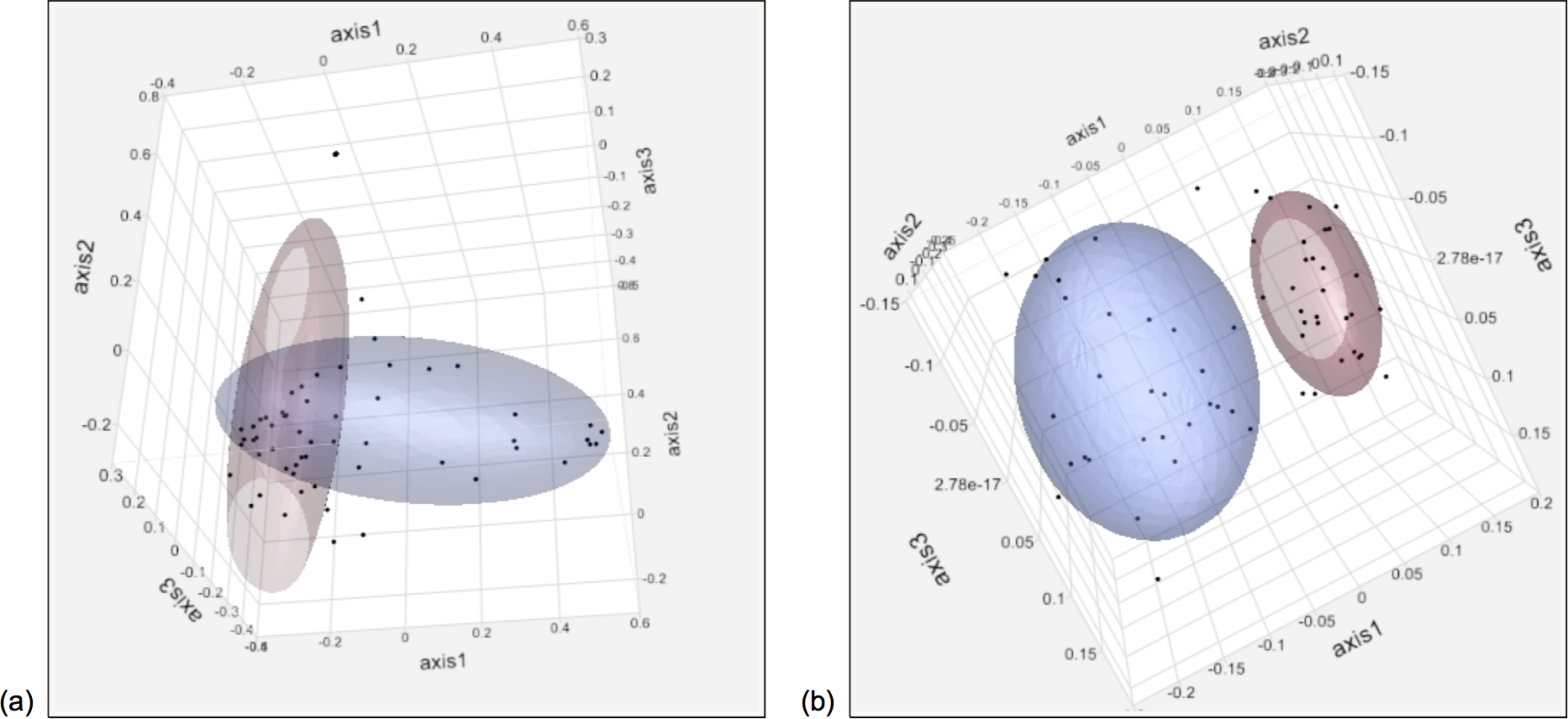
**Principal coordinate analysis (PCoA) of the Pre and 1Week time points for (a) plaque microbiota community structure, and (b) oral microbiota community membership (n = 30).** The plots denote 60% ellipsoid coverage and timepoints are represented by red (Pre), blue (1Week).

The alpha diversity measures for the plaque and oral microbiota are shown in S1 and S2 Figs. In both the plaque and oral microbiotas, Chao’s richness, Shannon’s evenness, and Inverse Simpson’s diversity all decreased after dental prophylaxis (*P* < 0.0001). Significant differences in community membership and structure between before and after dental prophylaxis are seen for both the plaque and oral microbiota (all unweighted UniFrac and AMOVA analyses *P* < 0.001, Fig 2).

Linear discriminant analysis effect size (LEfSe) identified genera that were enriched either prior to or one week after dental prophylaxis in both the plaque (128) and oral microbiota (113). Within the plaque environment, 13 genera had linear discriminant analysis (LDA) scores of ≥ 3.5, with six significantly enriched in the pre-treatment group, including *Streptobacillus* (4.08) and *Porphyromonas* (3.82) and seven afterwards, including *Pasteurella* (4.62) and *Neisseria* (4.29) (Table 1). From the oral microbiota, 18 genera had LDA scores of ≥ 3.5, with 17 prior to dental prophylaxis, including *Psychrobacter* (4.96) and only one–*Pseudomonas* (5.58)*–*after dental prophylaxis (Table 1).

**Table 1:** LEfSe (LDA score ≥ 3.5) identifying genera that were enriched in the plaque and oral microbiotas at the pre and one week post-dental prophylaxis timepoints (n = 30).

Plaque Microbiota				Oral Microbiota			
Pre		1Week		Pre		1Week	
Taxonomy	LDA Score	Taxonomy	LDA Score	Taxonomy	LDA Score	Taxonomy	LDA Score
*Streptobacillus*	4.08	*Pasteurella*	4.62	*Psychrobacter*	4.96	*Pseudomonas*	5.89
Unclassified Clostridiales	4.00	*Neisseria*	4.29	*Acinetobacter*	4.60		
*Porphyromonas*	3.86	*Pseudomonas*	4.07	*Treponema*	4.45		
*Desulfomicrobium*	3.74	*Actinomyces*	3.90	Unclassified Clostridiales	4.38		
*Desulfobulbus*	3.71	*Moraxella*	3.82	Unclassified Pasteurellaceae	4.31		
Unclassified Lachnospiraceae	3.52	Unclassified Moraxellaceae	3.80	*Porphyromonas*	4.12		
		Unclassified Actinomycetales	3.59	*Pasteurella*	4.10		
				*Actinomyces*	4.00		
				*Mannheimia*	3.75		
				Unclassified Peptostreptococcaceae	3.74		
				Unclassified Firmicutes	3.64		
				*Aerococcus*	3.62		
				*Haemophilus*	3.59		
				*Arcobacter*	3.58		
				SR1_genus_incertae _sedis	3.56		
				*Bibersteinia*	3.55		
				Unclassified Flavobacteriaceae	3.51		

### Effects of dental prophylaxis on the oral and plaque microbiotas of dogs over five weeks

#### Sequence quality

A random subsample of 10,858 sequences per sample was used to normalize the sequence numbers across all the study time points for the plaque samples, with Good’s coverage ranging from 80.8% to 97.3% (median = 94.1%). Similarly, a random subsample of 30,268 sequences per sample was used to normalize the sequence numbers for all the study time points for the oral samples, with a median Good’s coverage of 99.9%.

#### Relative abundance

**Fig 3:**
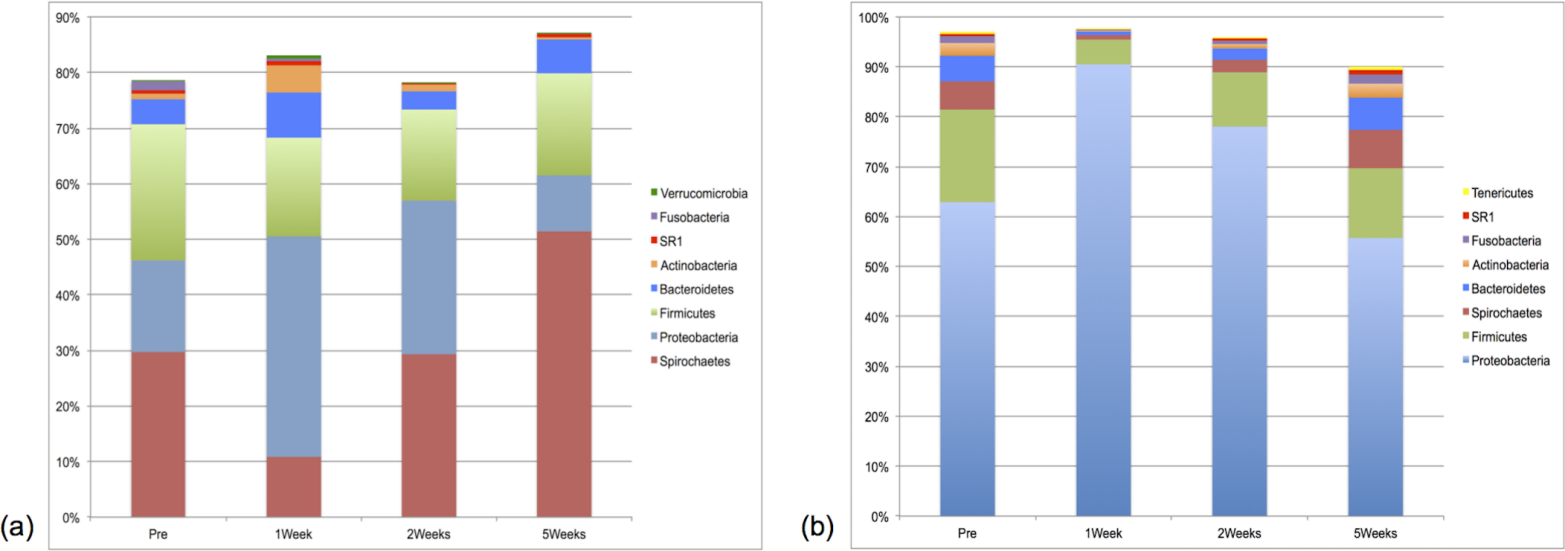
**Median percent relative abundances of bacterial phyla for (a) plaque and (b) oral microbiota samples (n = 10).** All study time points comparisons. Median relative abundance ≥ 0.5%. Refer to S3 Table for *P*-values.

Relative abundances of phyla across all study timepoints are depicted in Fig 3. Comparison of phyla at the various timepoints is reported in S3 Table. Relative abundance data from predominant genera are presented in S4 and S5 Tables.

Within the plaque microbiota, statistical differences in relative abundance were noted between time points for the putative periodontal pathogens *Porphyromonas* (*P* = 0.0451) and *Treponema* (*P* = 0.0150), with significantly higher abundances at the end of the study than compared to two weeks or one week after prophylaxis, respectively. *Pasteurella* had a higher relative abundance one week after dental prophylaxis compared to pre-treatment (*P* = 0.0451). Within the oral microbiota, statistical differences in relative abundance were seen across time points for several genera, including *Pseudomonas* and *Psychrobacter*, with higher abundances of *Pseudomonas* one and two weeks after dental prophylaxis compared to the start and endpoints of the study (*P* = 0.001 for all statistically significant comparisons) and lower relative abundances at those same time points for *Psychrobacter* (*P* < 0.002, for the Pre-1Week and Pre-2Weeks timepoint comparisons and *P* = 0.0451 for the 1Week-5Weeks timepoint comparison)

#### Alpha and beta diversity analyses

**Fig 4:**
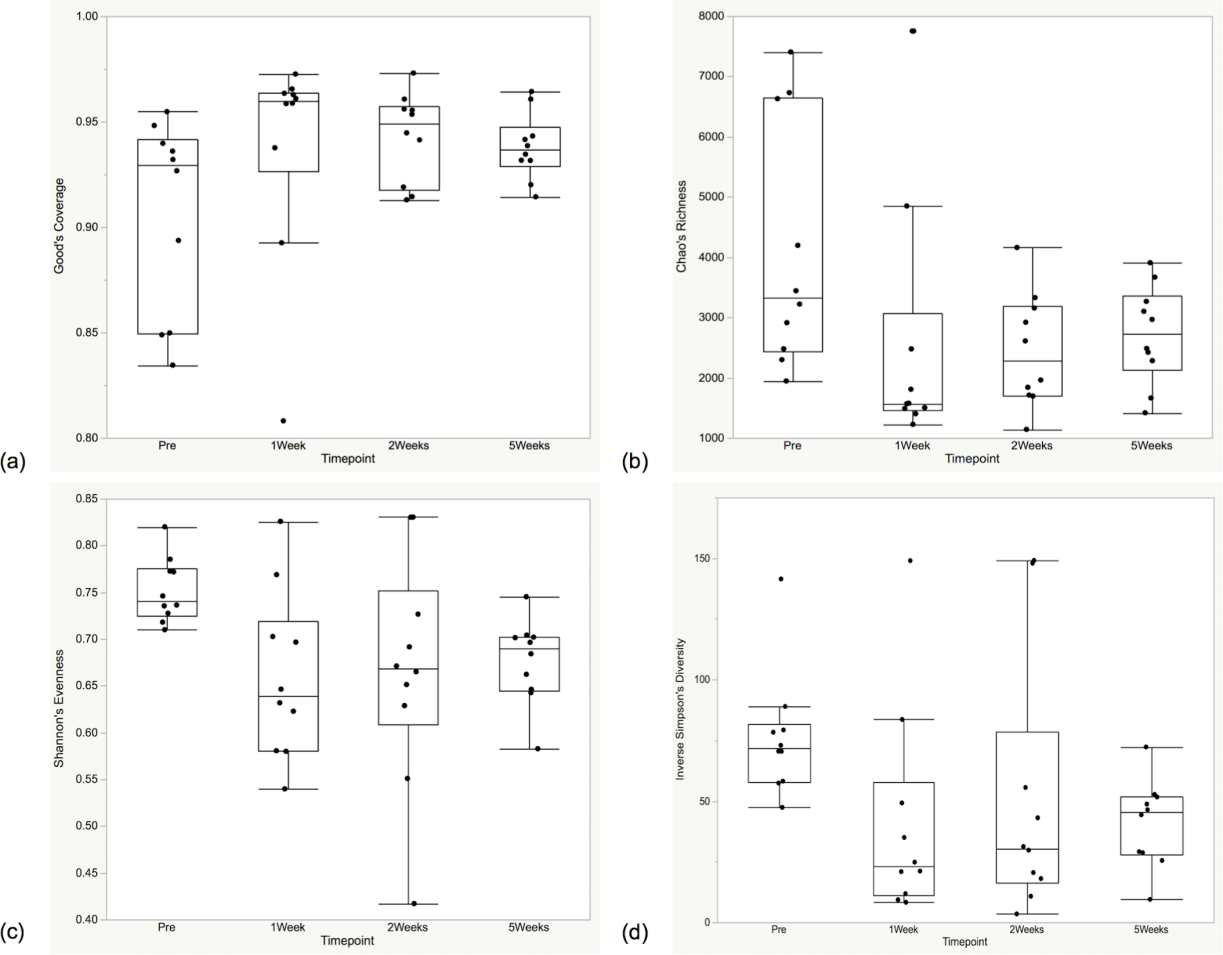
Plaque alpha diversity at all time points (n = 10). Quantile boxplots of (a) Good’s Coverage, (b) Chao’s Richness, (c) Shannon’s Evenness, and (d) Inverse Simpson’s Diversity. Significant differences were seen for parts (c) and (d) between the pre-prophylaxis time points and the end of the study (*P* < 0.01).

**Fig 5:**
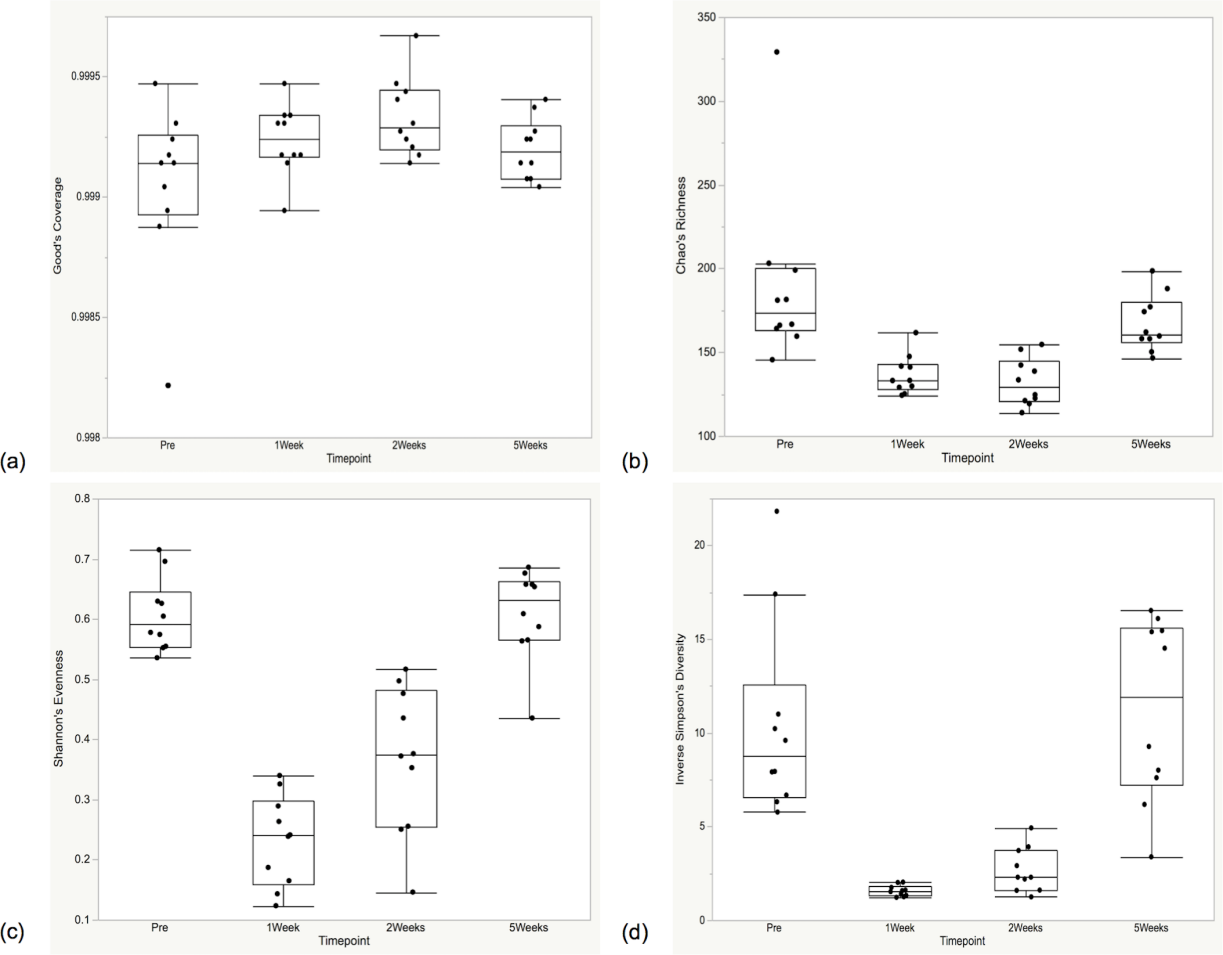
Oral microbiota alpha diversity at all time points (n = 10). Quantile boxplots of (a) Good’s Coverage, (b) Chao’s Richness, (c) Shannon’s Evenness, and (d) Inverse Simpson’s Diversity. Significant differences were seen for the Pre-1Week, Pre-2Weeks, 1Week-5Weeks, and 2Weeks-5Weeks time points comparisons, for (b), (c), and (d) (*P* < 0.006 for all comparisons).

**Fig 6:**
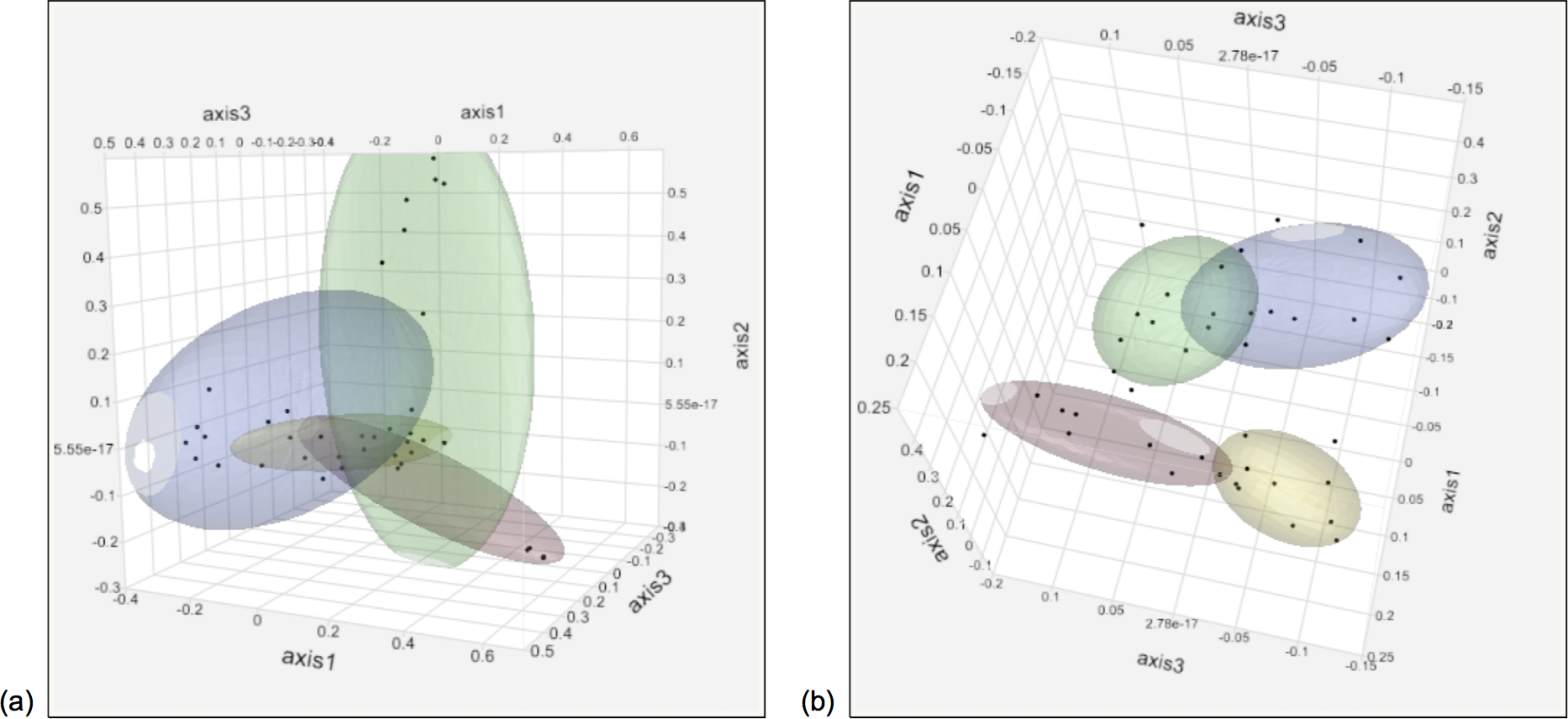
**Principal coordinate analysis (PCoA) of all time points for (a) plaque microbiota community structure, and (b) oral microbiota community membership (n = 10).** The plots denote 60% ellipsoid coverage and timepoints are represented by red (Pre), blue (1Week), green (2Weeks), and yellow (5Weeks).

The alpha diversity measures for the plaque and oral microbiotas are shown in Figs 4 and 5. Significant differences in community membership and structure were identified over time for both the plaque and oral microbiota (Fig 6, Table 2). In the plaque, there was a gradual return to the pre-dental state by the end of the study after a large disruption at the one week post-prophylaxis time point. In the oral microbiota, there was a distinct separation within the community membership and structure, with the Pre and 5Weeks time points clustering together and the 1Week and 2Weeks time points forming a separate cluster.

**Table 2:** Unweighted UniFrac test values for the Jaccard and the Yue and Clayton community index analyses of the plaque and oral microbiota (n = 10).

	Time Points Comparisons						
Site	Test	Pre-1Week	Pre-2Weeks	Pre-5Weeks	1Week-2Weeks	1Week-5Weeks	2Weeks-5Weeks
Plaque	Unweighted UniFrac–Jaccard	**0.002**	**0.05**	**0.02**	**0.022**	**0.015**	0.055
	Unweighted UniFrac–Yue and Clayton	**0.003**	**0.029**	**0.035**	0.07	0.275	0.077
Oral	Unweighted UniFrac–Jaccard	**0.016**	**0.024**	**0.017**	0.625	**0.017**	**0.021**
	Unweighted UniFrac–Yue and Clayton	**0.024**	**0.009**	**< 0.001**	0.097	**0.018**	**0.007**

### Comparison of the plaque and oral microbiotas prior to dental prophylaxis

#### Relative abundances

**Fig 7:**
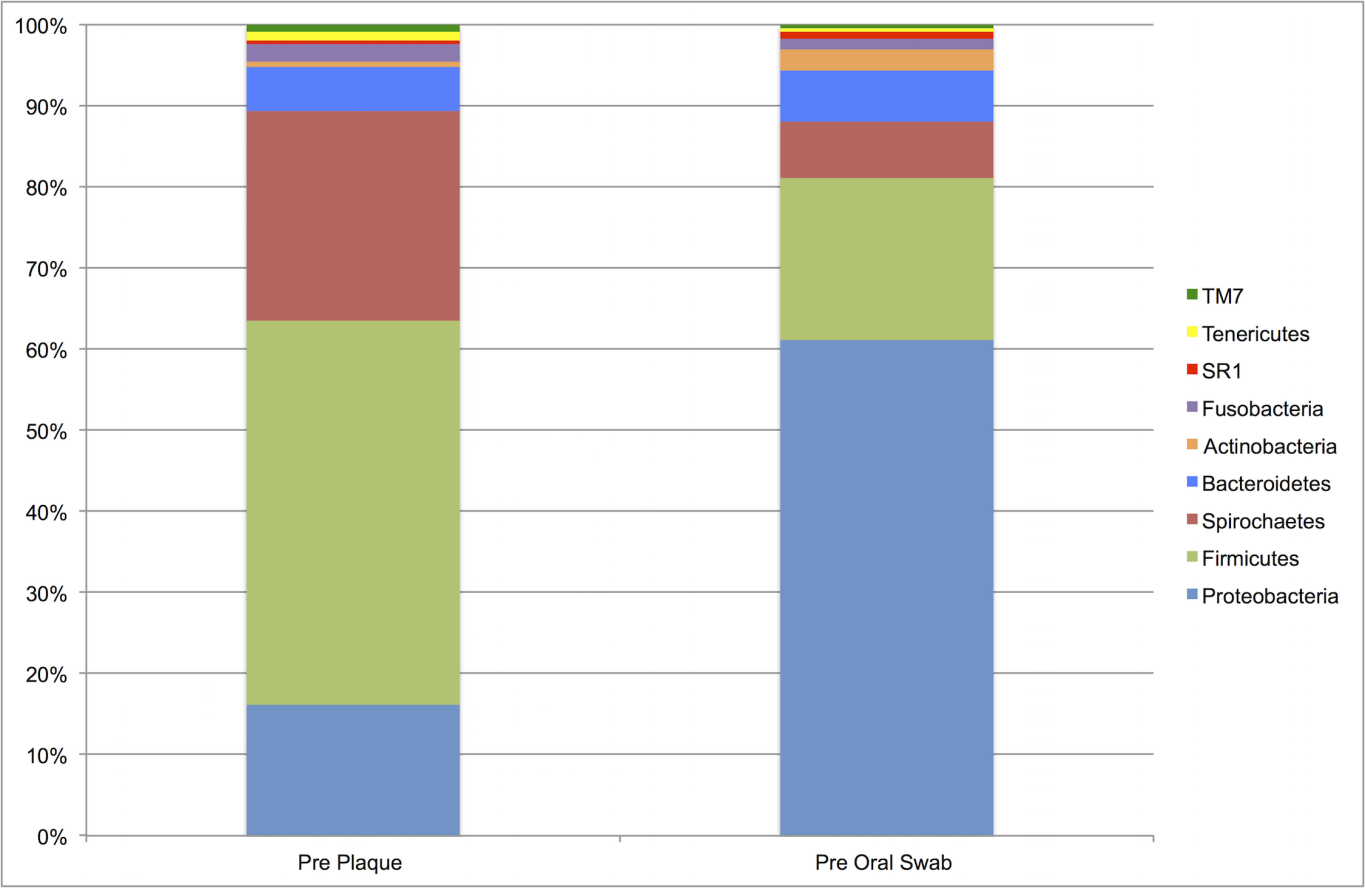
Pre-prophylaxis median percent relative abundances of bacterial phyla for plaque and oral microbiota samples (n = 30). Median relative abundance ≥ 0.5%.

Firmicutes (40.3%) and Spirochaetes (21.9%) predominated in the plaque environment, while Proteobacteria (58.3%) and Firmicutes (19.1%) predominated in samples from the oral microbiota (Fig 7). The differences in abundances for these aforementioned phyla were statistically significant (*P*_FDR_ < 0.0001).

**Fig 8:**
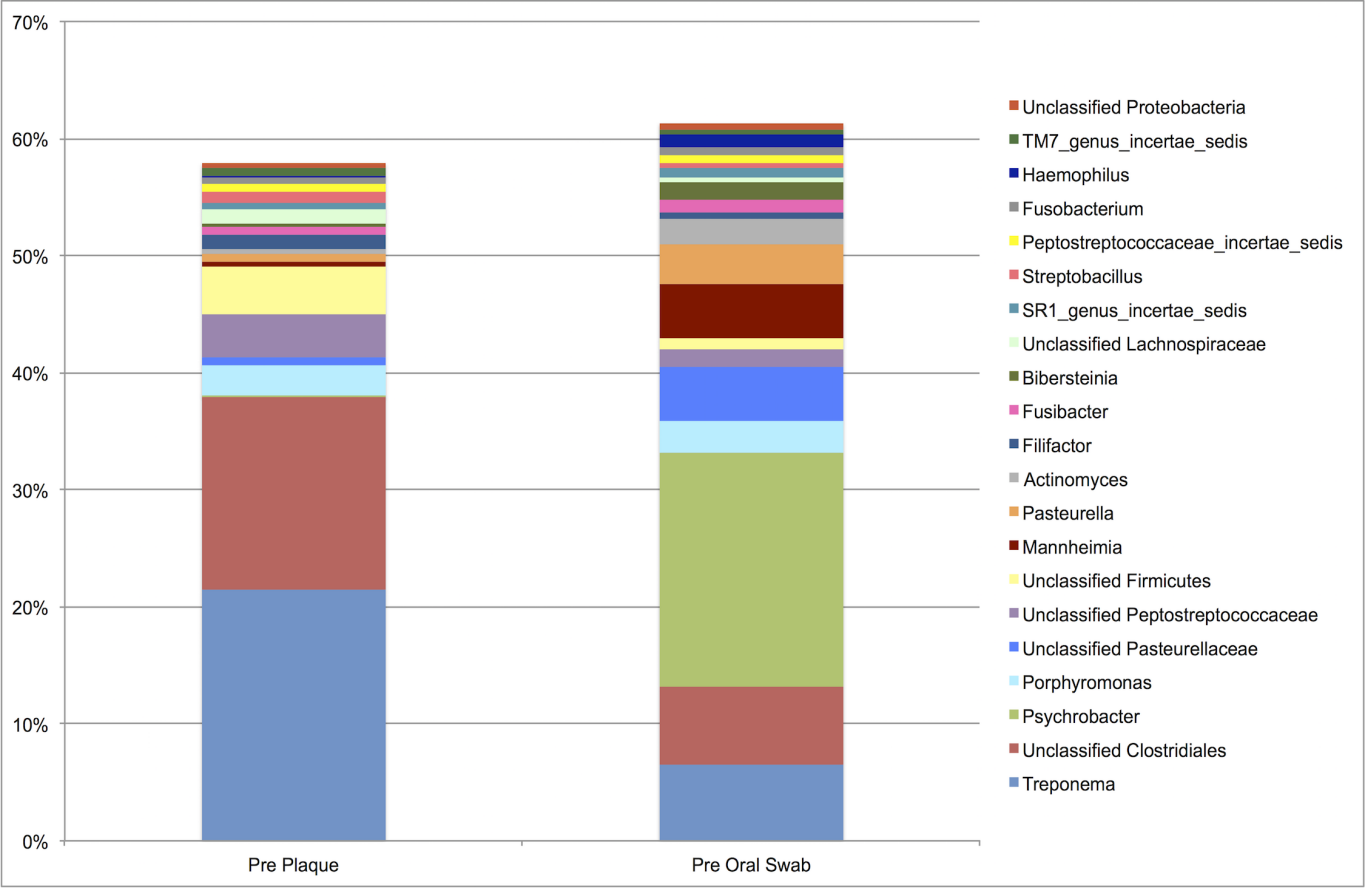
Pre-prophylaxis median percent relative abundances of bacterial genera for plaque and oral samples (n = 30). Median relative abundance ≥ 0.5%.

*Treponema* and an unclassified Clostridiales were highly abundant in both environments (Fig 8), but were significantly greater in plaque (*P*_FDR_ < 0.0001). A large number of genera differed significantly between plaque and oral microbiotas. An unclassified Firmicutes, and an unclassified Peptostreptococcaceae also had statistically significant differences in abundance from the oral samples (*P*_FDR_ < 0.0001). Within the oral microbiota, *Actinomyces*, *Mannheimia*, *Pasteurella*, *Psychrobacter*, and an unclassified Pasteurellaceae, all of which had statistically significant differences in abundance from the plaque samples (*P*_FDR_ < 0.0001). There was no statistical difference in the relative abundance of *Porphyromonas* between the plaque and oral microbiotas.

#### Alpha and beta diversity analyses

**Fig 9:**
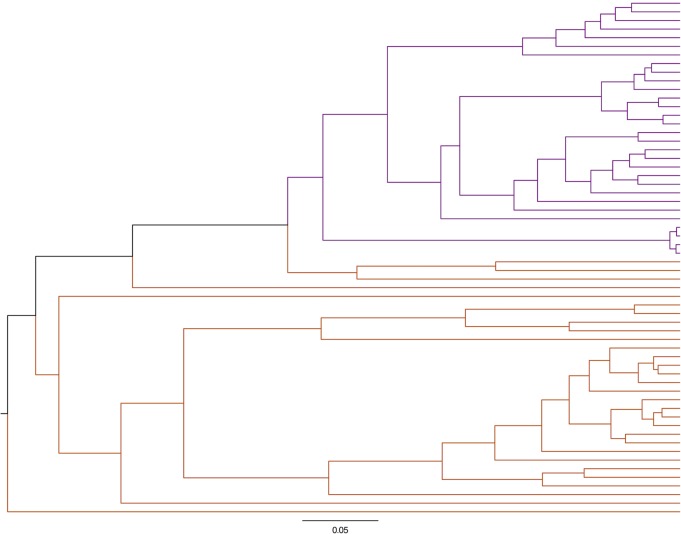
Yue and Clayton index dendrogram of bacterial structure (n = 30). Plaque samples (purple) and oral samples (orange).

Richness of plaque samples was significantly higher than oral samples (*P* = 0.0002), but there were no differences in diversity or evenness. Unweighted UniFrac tests as well as an AMOVA analysis demonstrate significant differences in both community membership and structure (*P* < 0.001) (Fig 9).

**Fig 10:**
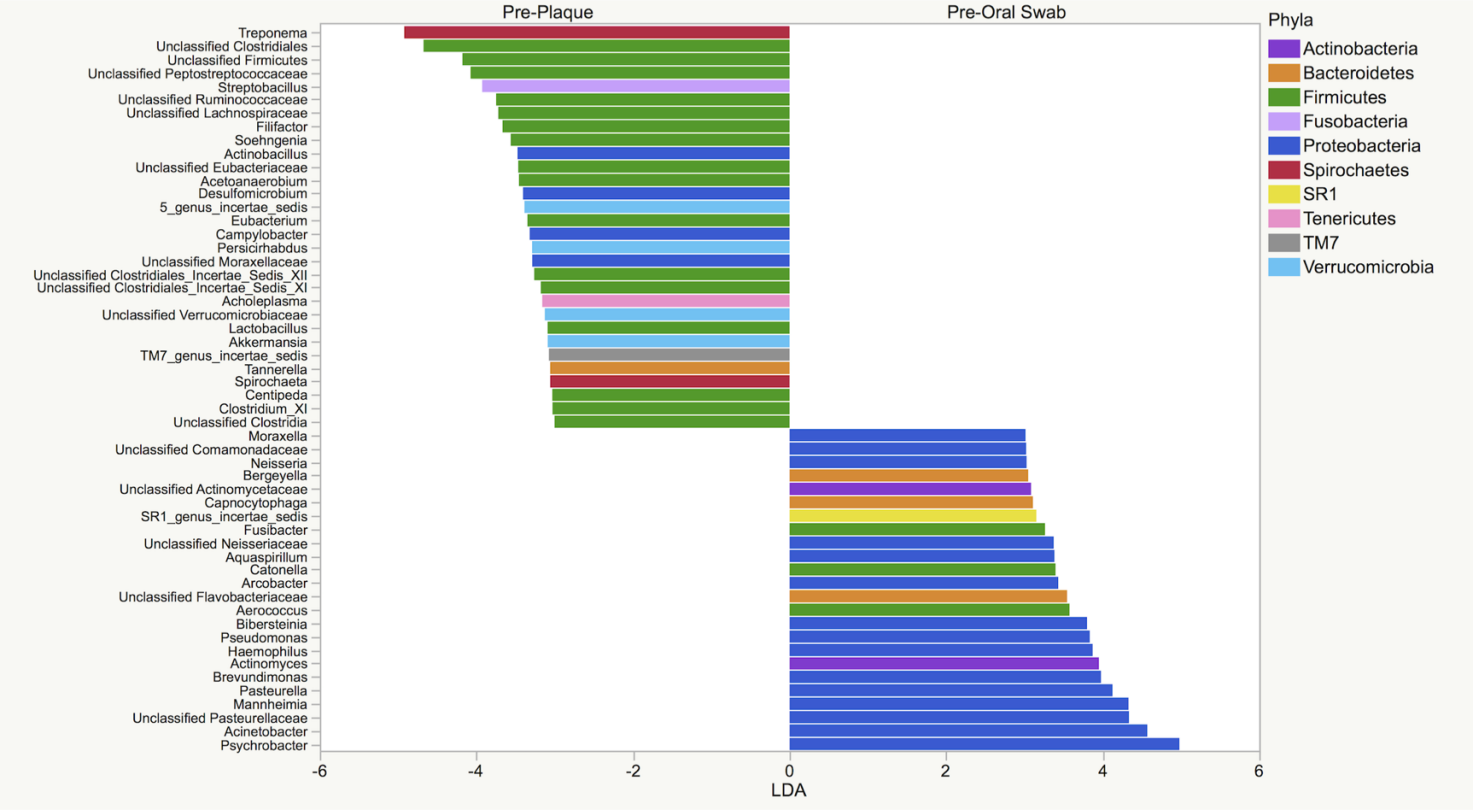
LEfSe results of plaque and oral microbiota samples of genera that were enriched prior dental prophylaxis (n = 30). LDA score ≥ 3.

LEfSe identified 95 genera that were significantly enriched in either environment. Fifty-four genera had linear discriminant analysis (LDA) scores of ≥ 3, with 30 in the plaque microbiota and 24 in the oral microbiota (Fig 10).

## Discussion

The canine oral cavity microbiota was highly rich and diverse, consistent with previous culture-independent studies [1–4,17], with significant differences based on sample type and a clear impact of dental prophylaxis. The variation between plaque and oral microbiota is consistent with a study on humans where the buccal mucosa, gingivae and hard palate had similar microbiotas while the saliva, tongue, tonsils and throat, and supra- and sub-gingival plaque each had distinctive communities [7]. This is not surprising based on the likelihood of different local microenvironments, but it highlights the complexity of the oral microbiota and the need to study specific environments. These results indicate that the oral microbiota is a poor (and potentially misleading) proxy for dental plaque.

Studies of microbiotas often involve a single sampling time, which precludes assessment of the degree of intra-individual variation and the impact of exogenous influences. Knowing how microbiotas change over time is crucial to understanding the effect of dental prophylaxis at both the individual and group levels. Longitudinal studies of other sample types in different species have often reported highly conserved results within the same individual [9,18–20]. Here, there were marked differences in different sample types (i.e. plaque vs. oral) and dental prophylaxis had a major impact on the two microbiotas.

Dental prophylaxis had dramatic effects on both the oral and plaque microbiotas, with pronounced changes in *Treponema*, *Pasteurella*, *Moraxella*, and *Neisseria*. Various treponemes, in particular *Treponema denticola* and *Treponema socranskii*, have been long-established as periodontal pathogens in dogs [21–23]. The significant decrease in *Treponema* (and more broadly, the phylum Spirochaetes) after dental prophylaxis may indicate a beneficial impact of the procedure. However, limitations in understanding of the pathophysiology of periodontal disease complicate clinical interpretation of these changes. Focusing on single pathogens or groups may also be suboptimal for what is likely a polymicrobial and complex disease. In contrast to the results for *Treponema*, the proportions of *Actinomyces*, *Corynebacterium*, and *Tannerella*, genera that have also been implicated as periodontal pathogens [1,24], increased in the first week after treatment. While seemingly incongruous with the dental prophylaxis having been recently performed, *Actinomyces*, *Corynebacterium* and *Tannerella* are slow growing and it is possible they were better able to proliferate with reduced bacterial competition. Additionally, *Actinomyces* and *Corynebacterium* have been shown to be early canine oral biofilm colonizing bacteria [5], and thus not surprising to see higher relative abundances one week post-prophylaxis. Ultimately, it is important to note these data are relative abundance, and it is possible the actual abundance of these taxa was decreased after dental prophylaxis.

While the oral microbiota changed after treatment, it did so in a different manner compared to plaque, with a dominance of *Pseudomonas* and the almost complete disappearance of the initially dominant *Psychrobacter* at the one week post-prophylaxis time point. The profound increase in one taxon not surprisingly impacted alpha diversity. When compared to other canine oral microbiota studies, the presence of *Pseudomonas* was somewhat unusual. *Pseudomonas* was found in a study that used molecular cloning-based sequencing of the 16S rRNA gene on bacterial isolates [24], wherein 30.9% of clones analysed were *Pseudomonas* sp. However, *Pseudomonas* was not identified in a different study of composite oral microbiota [17]. It is unlikely the high relative abundance of *Pseudomonas* was due to contamination during prophylaxis, as it is difficult to introduce exogenous bacteria and have them proliferate. *Pseudomonas* may play an as yet undiscovered role in the canine oral microbiota or its increase may simply reflect its ability to rapidly grow in a competition depleted environment, as can be reflected in increases in *Pseudomonas* in gut microbiota in disease or following antimicrobial administration [25,26]. Laboratory contamination is considered unlikely as pre-and post-treatment samples were processed together.

The high relative abundance of *Psychrobacter* was also somewhat unusual. Bacteria belonging to the genus *Psychrobacter* are psychrophilic or psychrotolerant and are typically found in cold environments. Beyond the environmental forms, little is known about *Psychrobacter* in humans, let alone dogs. In humans, *Psychrobacter* has been isolated from blood, cerebrospinal fluid, and various internal organs, and is considered to be a rare opportunistic pathogen [27]. One other study investigating the canine oral microbiota noted a presence in 1.3% (2/152) of clones, but no explanation was given [24]. In several studies on beef cattle, *Psychrobacter* was found to be a common nasopharyngeal inhabitant [28–30], but again, there was no discussion regarding its role and whether it is truly a commensal organism or an opportunistic pathogen. As with *Pseudomonas*, further investigation is warranted to better understand the role *Psychrobacter* plays in the canine oral microbiota.

Despite the major impact of dental prophylaxis, reversion towards the pre-treatment state occurred quickly for both the plaque and oral microbiota. The rapid return of the microbiota within five weeks to levels approximate to those seen pre-prophylaxis is analogous to what has been seen in the human gut following antibiotic insult [31], and suggests that both the plaque and oral microbiota are resilient. It also raises questions about the potential of dental prophylaxis to have beneficial clinical impacts through direct effects on the microbiota, if the microbiological changes are of short duration and dental prophylaxis is only performed intermittently.

*Porphyromonas* is one of the best-studied canine dental pathogens and interestingly, dental prophylaxis did not appear to have an effect on this genus, perhaps due in part to the fact the dogs included in the study had no overt signs of dental or oral disease. Nevertheless, there was a statistical difference between the two weeks and five weeks post-prophylaxis time points, with a significant increase at the latter timepoint. A limitation of current sequencing approaches is the lack of species-level resolution. There are numerous *Porphyromonas* species with different clinical relevance and the lack of change could have been as a result of one species of *Porphyromonas* increasing in relative abundance while others decreased. Alternatively, the stability could have been due to all *Porphyromonas* species maintaining steady relative abundances both prior to and one week after dental prophylaxis. Study of the impact of dental prophylaxis on individual species would be required to evaluate these results in greater detail.

Direct comparison of the pre-dental plaque and oral microbiotas yielded striking differences. At the genus level, the significant higher relative abundances of *Treponema* and an unclassified Clostridiales in the plaque, and significantly higher relative abundances of *Psychrobacter*, *Mannheimia*, and *Pasteurella* in the oral microbiota are consistent with the plaque microbiota harbouring greater populations of anaerobic and biofilm-associated taxa. Conversely, more aerobic and water-based taxa were observed in the oral samples.

Interestingly, richness was great in plaque, despite the composite nature of the oral samples, which sampled different oral sites (gums, tongue, and cheeks), each of which could have a somewhat different microbiota. This would be expected to increase richness of the composite sample. Furthermore, plaque is a highly specialized environment, and the richness and diversity of the microbiota in such environments is usually lower due to a few, highly adapted organisms predominating. The actual results of the study directly contrast with these notions, warranting further study, and in the interim, indicating the composition of, or changes in, the plaque microbiota cannot be accurately inferred through assessment of the more readily accessible oral microbiota.

Despite the advances in knowledge this research has provided, the study was not without its limitations. While there were several benefits to using colony dogs for the study, including ease of follow-up, consistency, and the ability to conduct a longitudinal study, there were inherent drawbacks to this choice. As the study subjects were all the same breed, health status, roughly the same age, and received the same food while housed in the same location, it is unclear if the results from the study could be extrapolated to a broader client-owned dog population. Additionally, the sample size, particularly the subsample of 10 dogs at the two and five weeks post-prophylaxis time points, was also a potential limitation, as it could have affected the power of the study.

## Conclusions

Dental prophylaxis had a major impact on the oral and plaque microbiotas, although changes were relatively short-term, with reversion populations akin to baseline within five weeks. Changes that were identified modify our understanding of the composition of these microbiotas and assessment of plaque provides insight into the microbial progression of early plaque (biofilm) development and the impact of a routine veterinary procedure, dental prophylaxis. Longitudinal study is important to help elucidate the pathophysiology of periodontal disease, a common and complex disease.

Comparison of plaque and oral samples provided additional insight. While plaque and oral samples are highly rich and diverse, there were significant differences, highlighting the need to study specific oral niches. Studies targeting plaque or plaque-associated diseases should use plaque samples, as oral samples are a poor proxy.

## Supporting information

S1 Fig Plaque microbiota alpha diversity at pre- and one week post-prophylaxis time points (n = 30). Quantile boxplots of (a) Good’s Coverage, (b) Chao’s Richness, (c) Shannon’s Evenness, and (d) Inverse Simpson’s Diversity. (*P* < 0.0001 for (b), (c), and (d)).
(TIFF)

S2 Fig Oral microbiota alpha diversity at pre- and one week post-prophylaxis time points (n = 30). Quantile boxplots of (a) Good’s Coverage, (b) Chao’s Richness, (c) Shannon’s Evenness, and (d) Inverse Simpson’s Diversity. (*P* < 0.0001 for (b), (c), and (d)).
(TIFF)

S1 Table Plaque microbiota genera percent relative abundances, *p-*values, and FDR *p-*values (n = 30). 54 selected genera from prior to, and one week post-dental prophylaxis, ordered by relative abundance.
(DOCX)

S2 Table Oral mirobiota genera percent relative abundances, *p-*values, and FDR *p-*values (n = 30). 54 selected genera from prior to, and one week post-dental prophylaxis, ordered by relative abundance.
(DOCX)

S3 Table Time points comparison *p-*values for phyla from plaque and oral samples collected at all study time points (n = 10). (DOCX)

S4 Table Plaque microbiota genera percent relative abundances, *p-*values, and FDR *p-*values (n = 10). 54 selected genera from all study time points, ordered by relative abundance.
(DOCX)

S5 Table Oral microbiota genera percent relative abundances, *p-*values, and FDR *p-*values (n = 10). 54 selected genera from all study timepoints, ordered by relative abundance.
(DOCX)
