# Supplementary figures and images for: Evaluation of the impact of dental prophylaxis on the oral microbiota of dogs
Source: PLoS One. 2018 Jun 25;13(6):e0199676. doi: 10.1371/journal.pone.0199676 (PMC6016910; doi:10.1371/journal.pone.0199676)

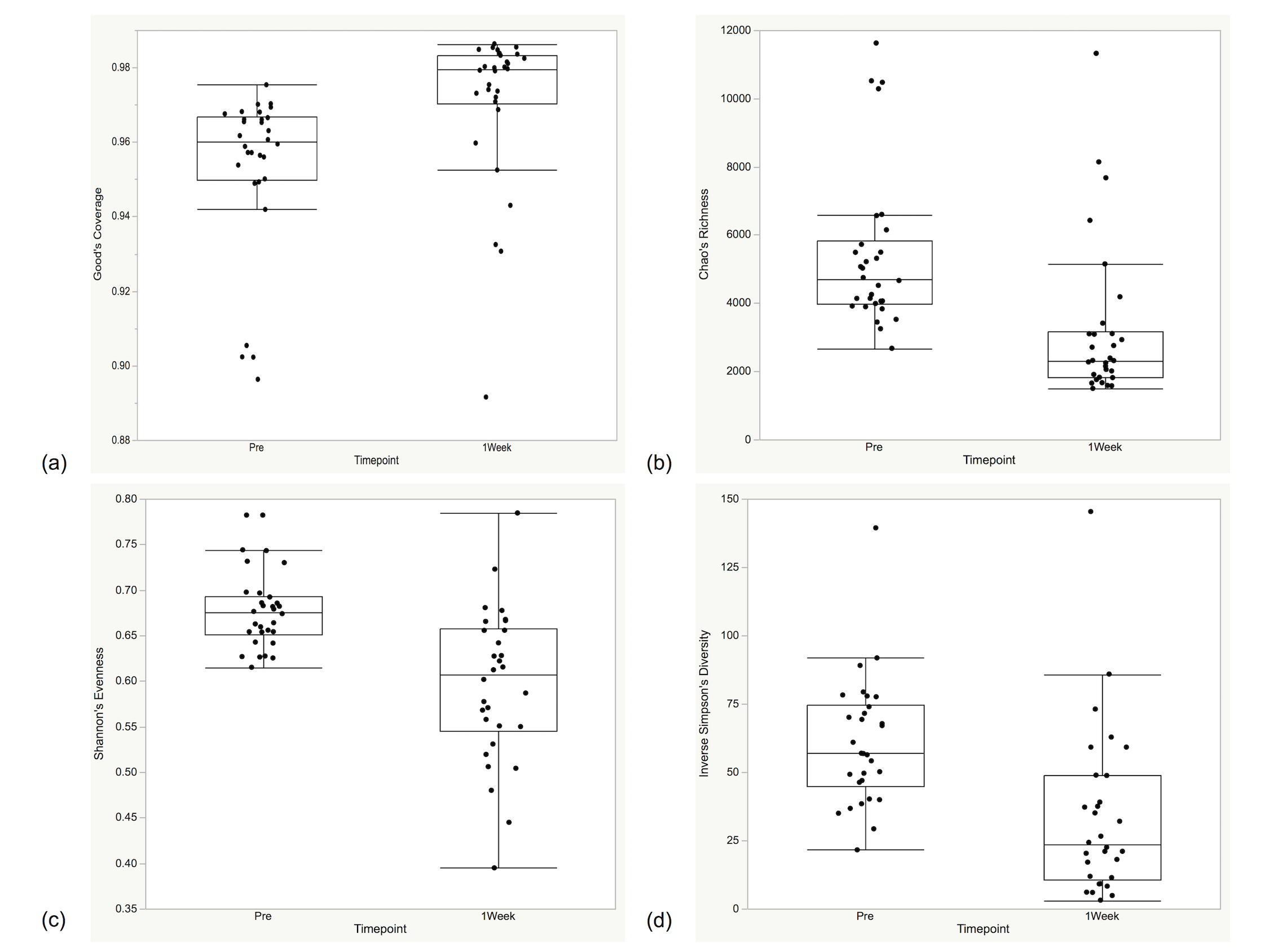

Supplement: S1 Fig — Quantile boxplots of (a) Good’s Coverage, (b) Chao’s Richness, (c) Shannon’s Evenness, and (d) Inverse Simpson’s Diversity. (P < 0.0001 for (b), (c), and (d)). (TIFF) [file pone.0199676.s001.tiff]

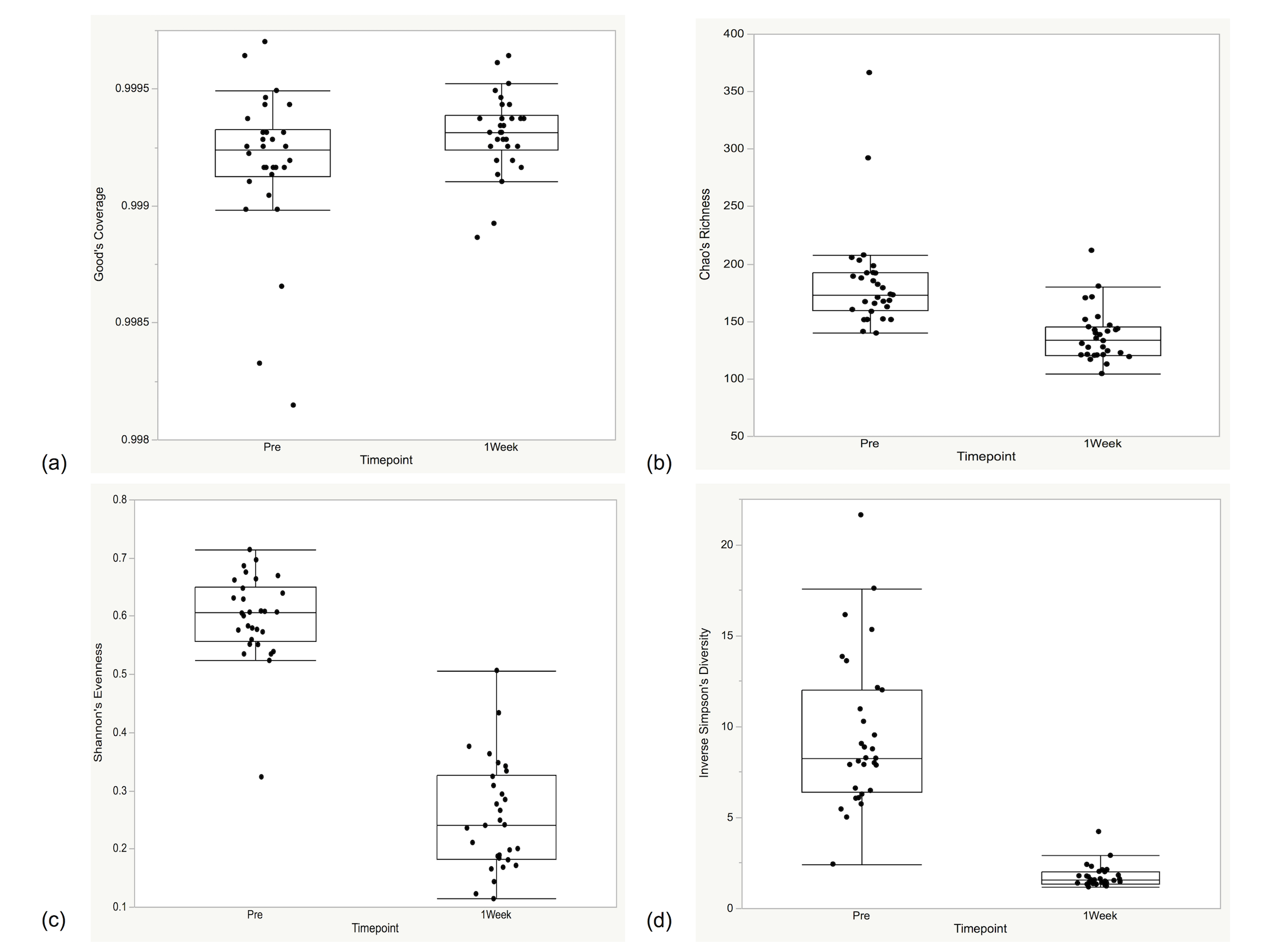

Supplement: S2 Fig — Quantile boxplots of (a) Good’s Coverage, (b) Chao’s Richness, (c) Shannon’s Evenness, and (d) Inverse Simpson’s Diversity. (P < 0.0001 for (b), (c), and (d)). (TIFF) [file pone.0199676.s002.tiff]
