# Supplementary material for: Evaluation of the impact of dental prophylaxis on the oral microbiota of dogs
Source: PLoS One. 2018 Jun 25;13(6):e0199676. doi: 10.1371/journal.pone.0199676 (PMC6016910; doi:10.1371/journal.pone.0199676)
Supplement: S1 Table — 54 selected genera from prior to, and one week post-dental prophylaxis, ordered by relative abundance. (DOCX) [file pone.0199676.s003.docx]

**S1 Table. Plaque microbiota genera percent relative abundances, *p-*values, and FDR *p-*values (n=30).** 54 selected genera from prior to, and one week post-dental prophylaxis, ordered by relative abundance.

| **Taxon** | **Pre-Dental Median %**  **(Min-Max)** | **1Week Median %**  **(Min-Max)** | ***p-*value** | **FDR *p-*value** |
| --- | --- | --- | --- | --- |
| *Treponema* | 32.33  (9.03 – 66.75) | 11.71  (1.99 – 63.11) | 0.0006 | 0.0035 |
| Unclassified Clostridiales | 9.01  (4.17 – 19.30) | 7.19  (0.92 – 16.06) | 0.0309 | 0.0644 |
| Unclassified Peptostreptococcaceae | 6.23  (0.23 – 20.27) | 5.20  (0.18 – 13.31) | 0.8809 | 0.8820 |
| *Porphyromonas* | 3.88  (0.51 – 15.40) | 3.24  (0.03 – 18.64) | 0.6370 | 0.6995 |
| Unclassified Lachnospiraceae | 1.86  (0.76 – 3.85) | 0.99  (0.13 – 3.60) | 0.0004 | 0.0031 |
| Unclassified Firmicutes | 1.41  (0.33 – 5.00) | 0.74  (0.07 – 5.27) | 0.0182 | 0.0410 |
| *Streptobacillus* | 1.34  (0.04 – 18.85) | 0.06  (0 – 10.98) | 0.0082 | 0.0263 |
| *Desulfomicrobium* | 1.11  (0.05 – 4.16) | 0.17  (0 – 1.45) | < 0.0001 | 0.0009 |
| *Pasteurella* | 1.11  (0.25 – 8.57) | 5.07  (0.01 – 39.73) | 0.0004 | 0.0031 |
| Unclassified Pasteurellaceae | 1.00  (0.21 – 7.69) | 1.38  (0.10 – 11.74) | 0.1168 | 0.1845 |
| *Acetoanaerobium* | 0.85  (0.04 – 3.12) | 0.47  (0.04 – 4.05) | 0.2854 | 0.3503 |
| SR1_genus_incertae_sedis | 0.78  (0.06 – 3.13) | 0.78  (0.01 – 2.79) | 0.7453 | 0.7892 |
| *Mannheimia* | 0.72  (0.04 – 4.59) | 0.70  (0.02 – 10.70) | 0.1572 | 0.2177 |
| Unclassified Proteobacteria | 0.72  (0.16 – 5.06) | 0.92  (0.13 – 2.64) | 0.4251 | 0.4782 |
| *Actinomyces* | 0.51  (0.07 – 4.14) | 2.07  (0.10 – 21.28) | 0.0022 | 0.0100 |
| Unclassified Comamonadaceae | 0.40  (0.01 – 2.81) | 1.06  (0.01 – 4.68) | 0.0366 | 0.0731 |
| *Neisseria* | 0.39  (0.04 – 2.65) | 2.09  (0.08 – 45.14) | 0.0117 | 0.0316 |
| *Desulfobulus* | 0.37  (0 – 8.12) | 0.03  (0 – 4.97) | 0.0101 | 0.0289 |
| *Bibersteinia* | 0.37  (0.03 – 1.45) | 0.51  (0 – 4.06) | 0.1449 | 0.2114 |
| *Campylobacter* | 0.37  (0.03 – 5.79) | 0.45  (0.03 – 1.33) | 0.6477 | 0.6995 |
| *Tannerella* | 0.36  (0.01 – 2.28) | 1.17  (0.03 – 3.43) | 0.0006 | 0.0035 |
| Unclassified Eubacteriaceae | 0.35  (0.01 – 2.28) | 0.38  (0.01 – 6.31) | 0.1512 | 0.2148 |
| *Moraxella* | 0.3  (0.03 – 2.13) | 1.01  (0.05 – 7.77) | 0.0013 | 0.0063 |
| Unclassified Neisseriaceae | 0.29  (0.01 – 2.73) | 0.38  (0.01 – 4.84) | 0.1200 | 0.1845 |
| *Catonella* | 0.25  (0.01 – 1.42) | 0.3  (0.01 – 2.50) | 0.8820 | 0.8820 |
| *Spirochaeta* | 0.24  (0 – 1.96) | 0.05  (0 – 1.90) | 0.1088 | 0.1781 |
| Unclassified Moraxellaceae | 0.21  (0.01 – 5.09) | 1.24  (0.03 – 9.79) | 0.0181 | 0.0410 |
| *Desulfovibrio* | 0.19  (0.02 – 1.89) | 0.03  (0 – 2.13) | 0.0083 | 0.0263 |
| Unclassified Spirochaetaceae | 0.19  (0 – 1.17) | 0.02  (0 – 0.83) | 0.0060 | 0.0213 |
| Unclassified Porphyromonadaceae | 0.17  (0 – 0.83) | 0.29  (0.02 – 0.97) | 0.1251 | 0.1876 |
| Unclassified Ruminococcaceae | 0.16  (0.02 – 8.54) | 0.18  (0.01 – 3.37) | 0.0310 | 0.0644 |
| Unclassified Bacteroidetes | 0.16  (0.05 – 1.87) | 0.11  (0.01 – 1.48) | 0.0740 | 0.1379 |
| Unclassified Deltaproteobacteria | 0.15  (0.01 – 3.62) | 0.07  (0.01 – 0.45) | 0.0102 | 0.0289 |
| *Paludibacter* | 0.13  (0 – 1.45) | 0.06  (0 – 0.69) | 0.1032 | 0.1742 |
| Unclassified Desulfovibrionaceae | 0.13  (0.04 – 0.54) | 0.02  (0 – 1.35) | 0.1912 | 0.2459 |
| *Capnocytophaga* | 0.12  (0 – 0.66) | 0.67  (0.01 – 3.51) | < 0.0001 | 0.0009 |
| *Arcobacter* | 0.12  (0 – 5.20) | 0.05  (0 – 1.44) | 0.0900 | 0.1568 |
| *Suttonella* | 0.12  (0 – 1.50) | 0.22  (0 – 6.46) | 0.0792 | 0.1425 |
| Unclassified Bacteriodales | 0.08  (0.02 – 0.65) | 0.08  (0 – 0.30) | 0.0522 | 0.1006 |
| Unclassified Betaproteobacteria | 0.08  (0.01 – 2.23) | 0.10  (0 – 3.12) | 0.8713 | 0.8820 |
| *Stenotrophomonas* | 0.08  (0.01 – 0.60) | 0.10  (0.01 – 0.92) | 0.3483 | 0.4088 |
| *Bergeyella* | 0.07  (0 – 1.22) | 0.12  (0 – 2.16) | 0.1749 | 0.2357 |
| *Haemophilus* | 0.07  (0.01 – 1.11) | 0.05  (0 – 0.53) | 0.2398 | 0.3012 |
| Unclassified Actinomycetales | 0.06  (0.02 – 0.37) | 0.67  (0.03 – 3.53) | < 0.0001 | 0.0001 |
| Unclassified Flavobacteriaceae | 0.06  (0.01 – 0.82) | 0.22  (0.05 – 3.51) | 0.0161 | 0.0395 |
| *Delftia* | 0.05  (0 – 0.43) | 0.08  (0 – 0.92) | 0.3704 | 0.4256 |
| Unclassified Actinomycetaceae | 0.04  (0 – 0.81) | 0.18  (0 – 1.33) | 0.0037 | 0.0143 |
| Unclassified Burkholderiales | 0.04  (0.01 – 0.30) | 0.24  (0.02 – 1.26) | < 0.0001 | 0.0009 |
| *Corynebacterium* | 0.04  (0 – 0.16) | 0.14  (0.02 – 1.33) | 0.0026 | 0.0110 |
| Unclassified Enterobacteriaceae | 0.03  (0 – 0.24) | 0.12  (0.01 – 0.83) | 0.0006 | 0.0035 |
| *Citrobacter* | 0.03  (0 – 0.34) | 0.14  (0.01 – 2.79) | 0.0138 | 0.0355 |
| *Acinetobacter* | 0.01  (0 – 1.22) | 0.18  (0 – 1.85) | 0.3239 | 0.3887 |
| *Pseudomonas* | 0  (0 – 0.36) | 0.08  (0 – 56.91) | 0.1789 | 0.2357 |
| *Aeromicrobium* | 0  (0 – 0.01) | 0.09  (0 – 0.73) | < 0.0001 | 0.0009 |
