# Supplementary material for: Evaluation of the impact of dental prophylaxis on the oral microbiota of dogs
Source: PLoS One. 2018 Jun 25;13(6):e0199676. doi: 10.1371/journal.pone.0199676 (PMC6016910; doi:10.1371/journal.pone.0199676)
Supplement: S2 Table — 54 selected genera from prior to, and one week post-dental prophylaxis, ordered by relative abundance. (DOCX) [file pone.0199676.s004.docx]

**S2 Table. Oral microbiota genera percent relative abundances, *p-*values, and FDR *p-*values (n=30).** 54 selected genera from prior to, and one week post-dental prophylaxis, ordered by relative abundance.

| **Taxon** | **Pre-Dental Median % (Min-Max)** | **1Week Median %**  **(Min-Max)** | ***p*-value** | **FDR *p*-value** |
| --- | --- | --- | --- | --- |
| *Pseudomonas* | 0.04  (0.02 – 34.45) | 79.72  (47.36 – 92.63) | < 0.0001 | < 0.0001 |
| *Psychrobacter* | 20.02  (0 – 40.00) | 0  (0 – 4.73) | < 0.0001 | < 0.0001 |
| Unclassified Pasteurellaceae | 6.50  (2.09 – 16.00) | 3.13  (0.61 – 8.05) | < 0.0001 | < 0.0001 |
| Unclassified Clostridiales | 6.76  (0.74 – 19.09) | 1.70  (0.48 – 7.40) | < 0.0001 | < 0.0001 |
| *Treponema* | 6.50  (0.53 – 12.03) | 0.99  (0.13 – 5.42) | < 0.0001 | < 0.0001 |
| *Pasteurella* | 3.49  (0.18 – 9.02) | 1.20  (0.13 – 3.69) | < 0.0001 | < 0.0001 |
| *Mannheimia* | 2.62  (0.42 – 9.53) | 1.86  (0.19 – 6.50) | 0.0479 | 0.0528 |
| *Porphyromonas* | 2.73  (0.51 – 7.01) | 0.25  (0.05 – 1.67) | < 0.0001 | < 0.0001 |
| *Bibersteinia* | 1.75  (0.55 – 3.95) | 1.03  (0.22 – 3.67) | 0.0025 | 0.0032 |
| *Actinomyces* | 2.16  (0.07 – 6.37) | 0.10  (0.02 – 1.10) | < 0.0001 | < 0.0001 |
| Unclassified Peptostreptococcaceae | 1.37  (0.24 – 3.59) | 0.26  (0.04 – 1.39) | < 0.0001 | < 0.0001 |
| *Haemophilus* | 1.06  (0.14 – 10.87) | 0.50  (0.09 – 3.06) | 0.0357 | 0.0410 |
| *Fusibacter* | 0.97  (0.02 – 2.11) | 0.33  (0.07 – 1.72) | < 0.0001 | < 0.0001 |
| Unclassified Neisseriaceae | 0.66  (0.09 – 3.44) | 0.35  (0.03 – 2.48) | 0.0399 | 0.0449 |
| Unclassified Firmicutes | 0.92  (0.14 – 2.48) | 0.07  (0.03 – 0.51) | < 0.0001 | < 0.0001 |
| *Fusobacterium* | 0.76  (0.21 – 1.45) | 0.20  (0.04 – 0.68) | < 0.0001 | < 0.0001 |
| SR1_genus_incertae_sedis | 0.84  (0.10 – 1.86) | 0.10  (0.02 – 1.01) | < 0.0001 | < 0.0001 |
| *Neisseria* | 0.60  (0.14 – 1.54) | 0.33  (0.09 – 0.96) | 0.0003 | 0.0005 |
| *Catonella* | 0.82  (0.05 – 1.47) | 0.08  (0.01 – 0.50) | < 0.0001 | < 0.0001 |
| Unclassified Proteobacteria | 0.57  (0.03 – 2.50) | 0.26  (0.03 – 0.74) | 0.0005 | 0.0007 |
| Unclassified Flavobacteriaceae | 0.66  (0.02 – 3.03) | 0.09  (0 – 0.99) | < 0.0001 | < 0.0001 |
| Unclassified Comamonadaceae | 0.50  (0.08 – 2.65) | 0.22  (0.08 – 1.07) | 0.0016 | 0.0021 |
| Peptostreptococcaceae_  incertae_sedis | 0.58  (0.18 – 2.63) | 0.12  (0.04 – 0.52) | < 0.0001 | < 0.0001 |
| *Filifactor* | 0.57  (0.22 – 1.68) | 0.07  (0.02 – 0.65) | < 0.0001 | < 0.0001 |
| *Arcobacter* | 0.57  (0.01 – 4.57) | 0.06  (0 – 0.84) | 0.0001 | 0.0002 |
| *Aquaspirillum* | 0.33  (0 – 2.14) | 0.26  (0 – 1.92) | 0.5253 | 0.5352 |
| TM7-genus_incertae_sedis | 0.44  (0.04 – 1.37) | 0.10  (0.03 – 0.23) | < 0.0001 | < 0.0001 |
| *Holdemania* | 0.39  (0.06 – 1.46) | 0.12  (0.01 – 0.65) | < 0.0001 | < 0.0001 |
| Unclassified Lachnospiraceae | 0.42  (0.08 – 1.84) | 0.05  (0.02 – 0.34) | < 0.0001 | < 0.0001 |
| *Streptobacillus* | 0.42  (0.04 – 1.28) | 0.05  (0 – 0.56) | < 0.0001 | < 0.0001 |
| Unclassified Clostridiales_  Incertae_Sedis_XII | 0.36  (0 – 1.90) | 0.10  (0.02 – 0.57) | 0.0016 | 0.0021 |
| *Capnocytophaga* | 0.33  (0.10 – 1.06) | 0.10  (0.02 – 0.36) | < 0.0001 | < 0.0001 |
| *Bergeyella* | 0.37  (0.02 – 0.94) | 0.05  (0 – 0.17) | < 0.0001 | < 0.0001 |
| Unclassified Prophyromonadaceae | 0.21  (0.03 – 0.76) | 0.04  (0.01 – 0.14) | < 0.0001 | < 0.0001 |
| *Kingella* | 0.24  (0.08 – 1.13) | 0.05  (0.02 – 0.21) | < 0.0001 | < 0.0001 |
| Unclassified Mollicutes | 0.23  (0.01 – 1.72) | 0.06  (0 – 0.27) | 0.0002 | 0.0003 |
| *Desulfomicrobium* | 0.27  (0.01 – 1.19) | 0.01  (0 – 0.19) | < 0.0001 | < 0.0001 |
| Unclassified Clostridiales_  Incertae_Sedis_XI | 0.17  (0 – 0.78) | 0.11  (0.01 – 0.36) | 0.0159 | 0.0190 |
| Unclassified Actinomycetaceae | 0.23  (0.01 – 1.25) | 0.03  (0 – 0.30) | < 0.0001 | < 0.0001 |
| *Suttonella* | 0.20  (0.04 – 0.85) | 0.04  (0.02 – 0.19) | < 0.0001 | < 0.0001 |
| *Helcococcus* | 0.21  (0.02 – 0.46) | 0  (0 – 0.14) | < 0.0001 | < 0.0001 |
| *Desulfobulbus* | 0.18  (0 – 1.53) | 0.01  (0 – 0.25) | < 0.0001 | < 0.0001 |
| Unclassified Burkholderiales | 0.11  (0 – 0.67) | 0.05  (0.01 – 0.21) | 0.0015 | 0.0021 |
| *Bacteroides* | 0.12  (0.01 – 0.30) | 0.02  (0.01 – 0.17) | < 0.0001 | < 0.0001 |
| *Wolinella* | 0.11  (0 – 1.05) | 0.04  (0 – 0.32) | 0.0017 | 0.0022 |
| *Conchiformibius* | 0.08  (0.02 – 0.38) | 0.06  (0.01 – 0.20) | 0.0726 | 0.0754 |
| *Tannerella* | 0.11  (0.03 – 0.46) | 0.02  (0 – 0.13) | < 0.0001 | < 0.0001 |
| Unclassified Moraxellaceae | 0.05  (0 – 0.43) | 0.07  (0 – 0.30) | 0.9546 | 0.9546 |
| *Parvimonas* | 0.08  (0.01 – 1.07) | 0.04  (0 – 0.22) | 0.0221 | 0.0259 |
| *Eubacterium* | 0.11  (0.01 – 0.47) | 0  (0 – 0.10) | < 0.0001 | < 0.0001 |
| Unclassified Lactobacillales | 0.11  (0 – 0.32) | 0.01  (0 – 0.08) | < 0.0001 | < 0.0001 |
| *Acinetobacter* | 0.06  (0.01 – 62.29) | 0.01  (0 – 0.42) | 0.0072 | 0.0089 |
| *Acholeplasma* | 0.01  (0 – 0.74) | 0  (0 – 0.30) | 0.0504 | 0.0544 |
| *Aerococcus* | 0.01  (0 – 11.59) | 0  (0 – 0.02) | 0.0718 | 0.0754 |
