# Supplementary material for: Evaluation of the impact of dental prophylaxis on the oral microbiota of dogs
Source: PLoS One. 2018 Jun 25;13(6):e0199676. doi: 10.1371/journal.pone.0199676 (PMC6016910; doi:10.1371/journal.pone.0199676)
Supplement: S3 Table — (DOCX) [file pone.0199676.s005.docx]

**S3 Table. Time points comparison *p-*values for phyla from plaque and oral samples collected at all study time points (n=10)**

|  | **Time Points Comparisons** | | | | | | |
| --- | --- | --- | --- | --- | --- | --- | --- |
| **Plaque Phyla** |  | Pre-1Week | Pre-2Weeks | Pre-5Weeks | 1Week-2Weeks | 1Week-5Weeks | 2Weeks-5Weeks |
|  | Actinobacteria | 0.0967 | 0.9419 | 0.4078 | 0.1600 | **0.0093** | 0.2164 |
|  | Bacteroidetes | 0.3643 | 0.8573 | 0.9758 | 0.0967 | 0.5967 | 0.4533 |
|  | Firmicutes | 0.2491 | 0.4078 | 0.1600 | 0.9610 | 0.8899 | 0.9610 |
|  | Fusobacteria | 0.1867 | **0.0150** | 0.0967 | 0.5967 | 0.9935 | 0.5967 |
|  | Proteobacteria | 0.1152 | 0.1152 | 0.8207 | 0.9935 | 0.0551 | 0.0807 |
|  | Spirochaetes | 0.2847 | 0.9935 | 0.3232 | 0.2164 | **0.0118** | 0.0967 |
|  | SR1 | 0.9995 | 0.6919 | 0.9995 | 0.5483 | 0.8573 | 0.0967 |
|  | Verrucomicrobia | 0.7374 | 0.8207 | 0.8899 | **0.0104** | 0.1867 | 0.9419 |
| **Oral Phyla** |  | Pre-1Week | Pre-2Weeks | Pre-5Weeks | 1Week-2Weeks | 1Week-5Weeks | 2Weeks-5Weeks |
|  | Bacteroidetes | **0.001** | **0.0093** | 0.9758 | 0.0967 | **0.0014** | **0.0093** |
|  | Firmicutes | **0.001** | **0.0072** | 0.4078 | **0.0366** | **0.0014** | 0.2491 |
|  | Fusobacteria | **0.001** | **0.0451** | 0.8207 | 0.2164 | **0.0025** | 0.0551 |
|  | Proteobacteria | **0.001** | **0.0237** | 0.8899 | **0.0296** | **0.001** | **0.0237** |
|  | Spirochaetes | **0.001** | 0.1362 | 0.5483 | 0.1362 | **0.001** | **0.0451** |
|  | SR1 | **0.0189** | 0.2847 | 0.5003 | 0.1362 | **0.0056** | **0.0093** |
|  | Tenericutes | **0.0451** | 0.3643 | 0.1867 | 0.3643 | **0.001** | **0.0056** |
|  | TM7 | **0.0033** | **0.0237** | 0.4078 | 0.5003 | **0.0033** | 0.0551 |
