# Supplementary material for: Evaluation of the impact of dental prophylaxis on the oral microbiota of dogs
Source: PLoS One. 2018 Jun 25;13(6):e0199676. doi: 10.1371/journal.pone.0199676 (PMC6016910; doi:10.1371/journal.pone.0199676)
Supplement: S4 Table — 54 selected genera from all study time points, ordered by relative abundance. (DOCX) [file pone.0199676.s006.docx]

**S4 Table. Plaque microbiota genera percent relative abundances, *p-*values, and FDR *p-*values (n=10).** 54 selected genera from all study time points, ordered by relative abundance. Superscripts denote statistical significances between timepoints, P ≤ 0.05.

| **Taxon** | **Pre-Dental Median % (Min-Max)** | **1Week Median % (Min-Max)** | **2Weeks Median % (Min-Max)** | **5Weeks Median % (Min-Max)** |
| --- | --- | --- | --- | --- |
| *Treponema* | 28.91 ^a,b^  (9.03 – 66.75) | 10.73 ^a^  (2.43 – 63.11) | 28.83 ^a,b^  (8.50 – 54.40) | 50.95 ^b^  (16.77 – 77.37) |
| Unclassified  Clostridiales | 10.64  (5.38 – 19.30) | 5.13  (1.78 – 16.06) | 7.61  (0.98 – 17.49) | 7.31  (4.09 – 12.49) |
| Unclassified Peptostreptococcaceae | 5.27  (0.25 – 7.82) | 2.50  (0.18 – 12.31) | 3.36  (0.37 – 15.62) | 5.41  (2.31 – 11.22) |
| Unclassified  Firmicutes | 2.73  (0.57 – 4.27) | 0.88  (0.36 – 2.06) | 0.56  (0.05 – 4.61) | 1.01  (0.44 – 2.85) |
| Unclassified Lachnospiraceae | 2.1  (0.78 – 3.85) | 0.86  (0.36 – 3.60) | 0.9  (0.05 – 6.53) | 2.03  (0.67 – 4.19) |
| *Porphyromonas* | 1.82 ^a,b^  (0.51 – 8.29) | 2.92 ^a,b^  (0.03 – 18.64) | 0.74 ^a^  (0.30 – 4.71) | 3.28 ^b^  (0.79 – 9.44) |
| *Streptobacillus* | 1.06 ^a^  (0.27 – 3.32) | 0.14 ^a,b,c^  (0.01 – 6.12) | 0.04 ^b^  (0 – 7.97) | 0.13 ^a,b,c^  (0.02 – 2.81) |
| Unclassified  Proteobacteria | 0.81  (0.25 – 3.55) | 1.10  (0.23 – 1.90) | 0.57  (0.02 – 2.19) | 0.47  (0.17 – 1.10) |
| SR1_genus_incertae_  sedis | 0.73  (0.06 – 2.23) | 0.81  (0.01 – 2.54) | 0.21  (0.07 – 1.64) | 0.54  (0.12 – 1.15) |
| *Actinomyces* | 0.63  (0.07 – 3.28) | 1.6  (0.10 – 5.74) | 0.77  (0.02 – 2.25) | 0.22  (0.04 – 5.02) |
| Unclassified Ruminococcaceae | 0.61  (0.03 – 8.26) | 0.27  (0.11 – 3.37) | 0.12  (0 – 10.48) | 0.23  (0.02 – 0.64) |
| *Acetoanaerobium* | 0.56  (0.04 – 2.55) | 0.29  (0.04 – 1.32) | 0.17  (0.02 – 0.68) | 0.48  (0.09 – 4.34) |
| *Pasteurella* | 0.51 ^a^  (0.25 – 5.62) | 9.04 ^b^  (0.01 – 32.38) | 0.74 ^a,b^  (0.09 – 6.30) | 1.41 ^a,b^  (0.10 – 30.41) |
| Unclassified  Eubacteriaceae | 0.5  (0.10 – 1.33) | 0.24  (0.02 – 6.15) | 0.23  (0 – 1.27) | 0.31  (0.06 – 0.91) |
| Unclassified  Pasteurellaceae | 0.45  (0.29 – 3.70) | 0.93  (0.10 – 11.74) | 2.09  (0.20 – 24.55) | 0.26  (0.01 – 5.04) |
| *Neisseria* | 0.43  (0.04 – 1.87) | 2.27  (0.19 – 21.13) | 0.75  (0.06 – 4.89) | 0.22  (0.02 – 1.72) |
| *Desulfomicrobium* | 0.25  (0.05 – 3.40) | 0.15  (0 – 1.45) | 0.09  (0 – 1.63) | 0.58  (0.03 – 4.48) |
| *Desulfovibrio* | 0.25  (0.02 – 1.54) | 0.02  (0 – 0.22) | 0.10  (0.01 – 3.53) | 0.44  (0.10 – 1.10) |
| *Desulfobulbus* | 0.25  (0 – 3.23) | 0.03  (0 – 0.88) | 0  (0 – 1.38) | 0.04  (0.01 – 0.19) |
| *Campylobacter* | 0.22  (0.05 – 5.79) | 0.45  (0.06 – 1.33) | 0.14  (0.01 – 0.52) | 0.08  (0 – 0.85) |
| Unclassified  Bacteroidetes | 0.2  (0.06 – 1.87) | 0.1  (0.02 – 0.96) | 0.03  (0.01 – 1.50) | 0.17  (0.05 – 0.29) |
| *Acinetobacter* | 0.2  (0 – 1.18) | 0.25  (0.09 – 1.85) | 0.06  (0 – 0.94) | 0.22  (0.07 – 0.80) |
| Unclassified Deltaproteobacteria | 0.17  (0.08 – 1.11) | 0.06  (0.01 – 0.20) | 0.03  (0.01 – 0.19) | 0.12  (0.04 – 0.65) |
| *Tannerella* | 0.16  (0.02 – 1.17) | 1.17  (0.03 – 3.43) | 0.19  (0 – 1.32) | 0.71  (0.21 – 1.24) |
| *Bibersteinia* | 0.15  (0.03 – 0.64) | 0.32  (0 – 2.09) | 1.02  (0.05 – 5.86) | 0.09  (0 – 0.95) |
| *Spirochaeta* | 0.15  (0 – 1.96) | 0.02  (0 – 0.62) | 0.1  (0 – 1.29) | 0.57  (0.06 – 4.72) |
| Unclassified Comamonadaceae | 0.13  (0.01 – 1.6) | 0.71  (0.01 – 2.78) | 0.44  (0.02 – 3.67) | 0.78  (0.10 – 3.49) |
| *Catonella* | 0.12  (0.01 – 0.85) | 0.27  (0.01 – 0.71) | 0.1  (0.02 – 0.49) | 0.22  (0.02 – 1.60) |
| Unclassified Desulfovibrionaceae | 0.12  (0.04 – 0.54) | 0.03  (0 – 0.10) | 0.13  (0.01 – 0.51) | 0.40  (0.11 – 1.94) |
| *Mannheimia* | 0.11  (0.04 – 1.40) | 0.71  (0.02 – 10.27) | 0.85  (0.03 – 13.20) | 0.11  (0 – 5.66) |
| Unclassified Porphyromonadaceae | 0.11  (0.01 – 0.48) | 0.48  (0.05 – 0.97) | 0.18  (0.03 – 0.34) | 0.22  (0.06 – 0.99) |
| *Acrobacter* | 0.1  (0 – 1.33) | 0.05  (0 – 1.44) | 0.04  (0 – 0.64) | 0.01  (0 – 0.09) |
| Unclassified Spirochaetaceae | 0.09  (0 – 0.57) | 0.01  (0 – 0.17) | 0.02  (0 – 0.31) | 0.06  (0.01 – 0.50) |
| Unclassified  Neisseriaceae | 0.09  (0.01 – 1.55) | 0.51  (0.01 – 2.80) | 0.24  (0 – 5.98) | 0.01  (0 – 0.28) |
| *Stenotrophomonas* | 0.08  (0.01 – 0.24) | 0.11  (0.03 – 0.92) | 0.13  (0 – 0.36) | 0  (0 – 0.02) |
| Unclassified Betaproteobacteria | 0.08  (0.01 – 1.55) | 0.11  (0.01 – 0.67) | 0.09  (0.03 – 0.87) | 0.01  (0 – 0.10) |
| Unclassified  Moraxellaceae | 0.08  (0.03 – 1.41) | 1.42  (0.03 – 4.10) | 0.47  (0.02 – 3.02) | 0.08  (0 – 9.92) |
| *Haemophilus* | 0.08  (0.04 – 1.11) | 0.18  (0.04 – 0.53) | 0.01  (0 – 0.49) | 0.01  (0 – 1.52) |
| *Moraxella* | 0.05 ^a^  (0.03 – 2.13) | 1.29 ^b^  (0.23 – 7.77) | 0.45 ^a,b,c^  (0.05 – 5.85) | 0.09 ^a,c^  (0 – 0.61) |
| *Paludibacter* | 0.05  (0 – 0.45) | 0.05  (0 – 0.24) | 0.04  (0 – 0.58) | 0.13  (0.01 – 1.32) |
| Unclassified Enterobacteriaceae | 0.05  (0 – 0.24) | 0.12  (0.03 – 0.83) | 0.05  (0.01 – 0.89) | 0.01  (0 – 0.02) |
| *Capnocytophaga* | 0.05 ^a^  (0 – 0.35) | 0.83 ^b^  (0.01 – 2.07) | 0.3 ^a,b^  (0.04 – 2.34) | 0.12 ^a,b^  (0.02 – 0.53) |
| Unclassified  Bacteroidales | 0.05  (0.02 – 0.26) | 0.06  (0 – 0.21) | 0.06  (0.01 – 0.12) | 0.09  (0.01 – 0.98) |
| *Suttonella* | 0.04  (0 – 0.53) | 0.22  (0 – 2.02) | 0.21  (0 – 1.11) | 0.04  (0 – 0.08) |
| Unclassified Actinomyceteaceae | 0.04  (0 – 0.81) | 0.16  (0 – 0.40) | 0.14  (0.01 – 0.53) | 0.05  (0 – 0.37) |
| *Corynebacterium* | 0.04 ^a^  (0 – 0.16) | 0.16 ^b^  (0.04 – 0.74) | 0.02 ^a,c^  (0 – 0.12) | 0.01 ^a,c^  (0 – 0.05) |
| Unclassified Flavobacteriaceae | 0.04  (0.01 – 0.34) | 0.46  (0.06 – 3.51) | 0.05  (0 – 5.33) | 0.01  (0 – 0.12) |
| Unclassified Actinomycetales | 0.04  (0.02 – 0.16) | 0.59  (0.03 – 2.34) | 0.19  (0.02 – 3.23) | 0.08  (0.02 – 0.38) |
| *Delftia* | 0.04  (0 – 0.18) | 0.09  (0 – 0.92) | 0.12  (0 – 0.59) | 0  (0 – 0) |
| *Citrobacter* | 0.03  (0 – 0.15) | 0.18  (0.01 – 2.79) | 0.06  (0 – 0.71) | 0  (0 – 0.01) |
| Unclassified Burkholderiales | 0.02  (0.01 – 0.29) | 0.1  (0.02 – 0.52) | 0.08  (0.02 – 1.12) | 0.06  (0 – 0.26) |
| *Bergeyella* | 0.02  (0 – 1.20) | 0.14  (0 – 2.16) | 0.1  (0.03 – 2.30) | 0.05  (0 – 0.22) |
| *Pseudomonas* | 0.01 ^a,c^  (0 – 0.26) | 0.19 ^a,b^  (0.01 – 24.91) | 6.88 ^b^  (0.01 – 52.40) | 0.01 ^a,c^  (0 – 0.07) |
| *Aeromicrobium* | 0 ^a^  (0 – 0.01) | 0.13 ^b^  (0 – 0.73) | 0 ^a,c^  (0 – 0.04) | 0 ^a,c^  (0 – 0) |
