# Supplementary material for: Evaluation of the impact of dental prophylaxis on the oral microbiota of dogs
Source: PLoS One. 2018 Jun 25;13(6):e0199676. doi: 10.1371/journal.pone.0199676 (PMC6016910; doi:10.1371/journal.pone.0199676)
Supplement: S5 Table — 54 selected genera from all study timepoints, ordered by relative abundance. (DOCX) [file pone.0199676.s007.docx]

**S5 Table. Oral mirobiota genera percent relative abundances, *p-*values, and FDR *p-*values (n=10).** 54 selected genera from all study timepoints, ordered by relative abundance. Superscripts denote statistical significances between timepoints, P ≤ 0.05.

| **Taxon** | **Pre-Dental Median % (Min-Max)** | **1Week Median % (Min-Max)** | **2Weeks Median % (Min-Max)** | **5Weeks Median % (Min-Max)** |
| --- | --- | --- | --- | --- |
| *Pseudomonas* | 0.05 ^a^  (0.02 – 34.45) | 80.26 ^b^  (69.69–90.54) | 65.65 ^b^  (41.56–89.65) | 0.01 ^a,c^  (0 – 0.91) |
| *Psychrobacter* | 20.26 ^a^  (0.01 – 40.00) | 0.01 ^b^  (0 – 0.03) | 0.01 ^b,c^  (0 – 0.04) | 0.02 ^a,c^  (0 – 53.01) |
| Unclassified Clostridiales | 7.28 ^a^  (3.79 – 12.70) | 2.44 ^b^  (0.67 – 3.44) | 4.44 ^a,b^  (1.21 – 10.80) | 5.39 ^a,c^  (3.05 – 10.32) |
| Unclassified Pasteurellaceae | 5.88 ^a^  (2.09 – 14.41) | 1.72 ^b^  (0.62 – 6.83) | 1.25 ^b^  (0.37 – 12.82) | 8.60 ^a,c^  (2.94 – 37.49) |
| *Treponema* | 5.60 ^a,d^  (3.60 – 12.03) | 0.98 ^b^  (0.18 – 2.42) | 2.30 ^a,b^  (0.48 – 8.07) | 7.41 ^c,d^  (3.42 – 18.51) |
| *Mannheimia* | 3.45 ^a,b,c^  (1.51 – 5.53) | 1.99 ^a,b^  (0.19 – 4.92) | 2.01 ^b^  (0.54 – 6.85) | 7.88 ^c^  (1.39 – 12.86) |
| *Pasteurella* | 3.56 ^a^  (1.24 – 9.02) | 1.31 ^b^  (0.45 – 3.69) | 2.17 ^a,b,c^  (1.01 – 5.89) | 3.41 ^a,c^  (1.39 – 10.73) |
| *Haemophilus* | 1.52 ^a^  (0.26 – 10.87) | 0.48 ^b^  (0.10 – 1.01) | 0.29 ^b^  (0.07 – 0.89) | 6.40 ^c^  (1.70 – 13.26) |
| *Porphyromonas* | 2.43 ^a^  (0.98 -7.01) | 0.29 ^b^  (0.05 – 0.63) | 1.02 ^b,c^  (0.05 – 1.90) | 3.13 ^a,c^  (0.28 – 5.11) |
| *Bibersteinia* | 1.77 ^a,b,c^  (0.68 – 2.81) | 1.29 ^a,b,c^  (0.36 – 2.27) | 0.72 ^a,b^  (0.15 – 3.66) | 2.34 ^a,c^  (0.87 – 19.20) |
| *Actinomyces* | 2.16 ^a^  (0.98 – 6.37) | 0.12 ^b^  (0.02 – 0.57) | 0.67 ^a,c^  (0.05 – 2.64) | 2.19 ^a,c^  (0.30 – 3.39) |
| *Moraxella* | 0.40  (0.21 – 1.03) | 0.60  (0.19 – 0.84) | 0.51  (0.30 – 2.77) | 1.77  (0.32 – 3.95) |
| Unclassified Peptostreptococcaceae | 1.32  (0.63 -3.08) | 0.26  (0.08 – 0.65) | 0.87  (0.22 – 2.29) | 0.77  (0.33 – 1.81) |
| *Fusibacter* | 1.22 ^a^  (0.44 – 2.11) | 0.24 ^b^  (0.07 – 1.09) | 0.90 ^a,c^  (0.25 – 1.55) | 0.54 ^a,b,c^  (0.15 – 3.72) |
| *Fusobacterium* | 0.91 ^a^  (0.62 – 1.45) | 0.26 ^b^  (0.06 – 0.53) | 0.63 ^a,b,c^  (0.18 – 1.23) | 0.56 ^a,c^  (0.19 – 1.23) |
| *Neisseria* | 0.62  (0.24 – 1.54) | 0.41  (0.13 – 0.59) | 0.91  (0.22 – 2.12) | 0.24  (0.11 – 2.35) |
| *Catonella* | 0.64  (0.19 – 1.21) | 0.09  (0.02 – 0.27) | 0.34  (0.02 – 0.82) | 0.89  (0.19 – 1.76) |
| SR1_genus_incertae_  sedis | 0.58  (0.25 – 1.76) | 0.08  (0.02 – 1.01) | 0.32  (0.04 – 1.06) | 0.95  (0.41 – 2.12) |
| *Arcobacter* | 0.39  (0.06 – 4.57) | 0.06  (0.01 – 0.84) | 0.11  (0.03 – 0.74) | 1.29  (0.04 – 8.29) |
| Peptostreptococcaceae_  incertae_sedis | 0.64  (0.30 – 0.87) | 0.13  (0.07 – 0.24) | 0.50  (0.12 – 1.03) | 0.49  (0.19 – 1.87) |
| Unclassified Flavobacteriaceae | 0.40  (0.02 – 1.63) | 0.06  (0 – 0.99) | 0.11  (0.01 – 1.76) | 1.13  (0.12 – 2.99) |
| Unclassified Proteobacteria | 0.47  (0.20 – 2.50) | 0.16  (0.03 – 0.74) | 0.43  (0.08 – 2.09) | 0.56  (0.13 – 1.97) |
| Unclassified Firmicutes | 0.90  (0.39 – 2.48) | 0.07  (0.03 – 0.31) | 0.17  (0.07 – 0.76) | 0.37  (0.11 – 1.14) |
| *Streptobacillus* | 0.44 ^a^  (0.09 – 0.86) | 0.05 ^b^  (0.01 – 0.12) | 0.03 ^b^  (0 – 0.15) | 0.91 ^a,c^  (0.19 – 3.98) |
| *Filifactor* | 0.64  (0.36 – 1.68) | 0.08  (0.02 – 0.13) | 0.20  (0.09 – 0.63) | 0.36  (0.12 – 0.70) |
| Unclassified Lachnospiraceae | 0.42  (0.23 – 0.80) | 0.07  (0.02 – 0.12) | 0.11  (0.03 – 0.32) | 0.48  (0.31 – 1.28) |
| Unclassified Neisseriaceae | 0.44  (0.09 – 3.44) | 0.17  (0.04 – 0.89) | 0.22  (0.06 – 0.65) | 0.19  (0.11 – 1.03) |
| *Holdemania* | 0.39  (0.13 – 1.30) | 0.10  (0.01 – 0.65) | 0.27  (0.03 – 1.24) | 0.18  (0.06 – 0.74) |
| Unclassified Prophyromonadaceae | 0.31  (0.12 – 0.57) | 0.05  (0.02 – 0.14) | 0.10  (0.01 – 0.22) | 0.45  (0.12 – 0.81) |
| Unclassified Clostridiales  _Incertae_Sedis_XII | 0.41  (0.11 – 1.04) | 0.09  (0.02 – 0.49) | 0.16  (0.12 – 0.36) | 0.22  (0.03 – 0.53) |
| TM7_genus_incertae_  sedis | 0.44  (0.13 – 0.99) | 0.08  (0.03 – 0.19) | 0.12  (0.07 – 0.53) | 0.23  (0.15 – 0.60) |
| Unclassified Moraxellaceae | 0.07  (0.01 – 0.23) | 0.08  (0 – 0.21) | 0.10  (0.03 – 1.30) | 0.60  (0.03 – 3.96) |
| Unclassified Mollicutes | 0.29  (0.01 – 0.90) | 0.03  (0.01 – 0.15) | 0.05  (0 – 0.69) | 0.48  (0.04 – 2.27) |
| *Capnocytophaga* | 0.30 ^a^  (0.17 – 1.06) | 0.08 ^b^  (0.02 – 0.29) | 0.21 ^a,b^  (0.06 – 0.81) | 0.18 ^a,b^  (0.05 – 0.55) |
| Unclassified Actinomycetaceae | 0.23  (0.09 – 0.97) | 0.03  (0 – 0.15) | 0.12  (0.01 – 0.45) | 0.35  (0.08 – 1.04) |
| *Bergeyella* | 0.23  (0.02 – 0.94) | 0.02  (0 – 0.17) | 0.06  (0.01 – 0.33) | 0.25  (0.10 – 1.37) |
| *Desulfomicrobium* | 0.30  (0.12 – 1.19) | 0.01  (0 – 0.06) | 0.03  (0 – 0.11) | 0.13  (0.01 – 0.42) |
| *Suttonella* | 0.20  (0.04 – 0.56) | 0.05  (0.02 – 0.09) | 0.10  (0.07 – 0.18) | 0.07  (0.01 – 0.34) |
| *Helcococcus* | 0.21  (0.05 – 0.39) | 0.01  (0 – 0.05) | 0.01  (0 – 0.06) | 0.15  (0.02 – 1.19) |
| *Kingella* | 0.12  (0.08 – 0.43) | 0.03  (0.02 – 0.21) | 0.06  (0.02 – 0.36) | 0.15  (0.02 – 0.81) |
| *Acetoanaerobium* | 0.24  (0.12 – 0.63) | 0.01  (0 – 0.07) | 0.03  (0.01 – 0.32) | 0.07  (0 – 0.27) |
| *Centipeda* | 0.13  (0.06 – 2.08) | 0.02  (0 – 0.17) | 0.06  (0.01 – 0.15) | 0.15  (0 – 0.80) |
| *Bacteroides* | 0.11  (0.04 – 0.28) | 0.02  (0.01 – 0.07) | 0.06  (0 – 0.21) | 0.11  (0.02 – 0.30) |
| Unclassified Lactobacillales | 0.09  (0.04 – 0.24) | 0.01  (0 – 0.03) | 0  (0 – 0.03) | 0.19  (0.02 – 0.38) |
| *Eubacterium* | 0.18  (0.01 – 0.30) | 0.01  (0 – 0.02) | 0.01  (0 – 0.03) | 0.10  (0.05 – 0.42) |
| Unclassified Burkholderiales | 0.10  (0.04 – 0.39) | 0.04  (0.01 – 0.10) | 0.07  (0.01 – 0.23) | 0.06  (0 – 0.15) |
| *Conchiformibius* | 0.07  (0.03 – 0.15) | 0.06  (0.01 – 0.14) | 0.03  (0 – 0.21) | 0.10  (0.08 – 7.60) |
| *Tannerella* | 0.11 ^a^  (0.04 – 0.46) | 0.02 ^b^  (0 – 0.04) | 0.06 ^a,b^  (0.01 – 0.22) | 0.06 ^a,b^  (0.01 – 0.15) |
| Unclassified Bacteroidetes | 0.13  (0.04 – 0.68) | 0.01  (0 – 0.02) | 0.02  (0 – 0.09) | 0.07  (0 – 0.16) |
| Unclassified Bacteroidales | 0.10  (0.03 – 0.19) | 0.02  (0 – 0.06) | 0.04  (0 – 0.09) | 0.07  (0.01 – 0.36) |
| *Spirochaeta* | 0.07 ^a,d^  (0.02 – 0.31) | 0.01 ^b^  (0 – 0.05) | 0.02 ^a,b^  (0 – 0.12) | 0.12 ^a,c^  (0.02 – 0.26) |
| *Desulfobulbus* | 0.019  (0.03 – 1.31) | 0.01  (0 – 0.05) | 0  (0 – 0.08) | 0.01  (0 – 0.09) |
| *Wolinella* | 0.05  (0.01 – 0.89) | 0  (0 – 0.32) | 0.02  (0 – 0.14) | 0.08  (0 – 0.33) |
| *Acinetobacter* | 0.06  (0.01 – 19.96) | 0.01  (0 – 0.03) | 0.01  (0 – 0.01) | 0  (0 – 0.13) |
